# Based on two coconut (*Cocos nucifera* L.) genome-wide investigation of NODULE-INCEPTION-like protein family: evolution and expression profiles during development and stress

**DOI:** 10.3389/fpls.2025.1565559

**Published:** 2025-04-16

**Authors:** Yubin Li, Yujie Li, Xue Hai, Cici Bao, Jinyang Zhao, Shouchuang Wang, Xianming Zhou, Penghui Liu, Chengcheng Si

**Affiliations:** ^1^ Sanya Institute of Breeding and Multiplication, School of Tropical Agriculture and Forestry, Hainan University, Haikou, China; ^2^ Hainan Seed Industry Laboratory, Sanya, China

**Keywords:** NLP transcription factors, coconut, phylogenetic analysis, abiotic stress, gene expression profiles

## Abstract

NODULE-INCEPTION-Like Protein (NLP) is a plant-specific transcription factor that plays a crucial role in nitrate uptake and stress responses. However, studies of *NLP* gene family in coconut are lacking. In this study, 7 *NLP* genes in the *Aromatic Dwarf* and 6 *NLP* genes in *Hainan Tall* coconut varities were identified. Furthermore, protein interaction network analysis suggested that CnD-NLP3/4/5 and CnT-NLP3/4/5 potentially interact with NRG2, HRS1, and LBD37, which may significantly influence nitrogen utilization in coconut, analysis of the promoters of *CnD-NLPs* and *CnT-NLPs* revealed many cis-acting elements associated with abiotic stress responses. Additionally, expression profiling showed that *CnD-NLPs* and *CnT-NLPs* were mainly expressed in mature leaves. Moreover, qRT-PCR analysis demonstrated that all identified *CnD-NLPs* responded to nitrogen deficiency, drought, and salt stress. Specifically, *CnD-NLP5* may be a potentially critical gene in nitrogen deficiency responses, *CnD-NLP4/5/6* and *CnD-NLP1* may play a pivotal role in drought and salt stress response, respectively. And screening of homologous genes showed that *CnD-NLP5* and *AtNLP8*, *CnD-NLP4* and *AtNLP7*, *CnD-NLP1* and *OsNLP2* are homologous genes, with similar functions, further supporting their involvement in nitrogen deficiency, drought, and salt stress responses. These results lay the foundation for future research on the *NLPs* function and potential applications in coconut.

## 1 Introduction

The coconut (*Cocos nucifera* L., 2n=32), a member of the Palmaceae family, is an important perennial tropical fruit and oil crop, often revered as the “tree of life” due to its extensive economic value (Beveridge et al., 2022). Thriving in tropical coastal regions, coconut trees are primarily categorized into two types-dwarf and tall-based on their morphological and growth characteristics (Beveridge et al., 2022; Mat et al., 2022).

Tall coconuts, which can grow up to 20-30 meters, are slower to mature, typically bearing fruit after 6-10 years (Harries, 1978; DebMandal and Mandal, 2011). In contrast, dwarf coconuts, known for their rapid growth, reach heights of 10-15 meters and start bearing numerous small nuts within just 4-5 years (Swaminathan and Nambiar, 1961). This early productivity makes them a preferred choice for breeding new varieties (Xiao et al., 2017). However, as global climate change, coconut palms are facing increasing challenges from abiotic stresses such as nitrogen deficiency, drought, and salt stress. These stresses severely impact their growth and productivity (Sousa Santos et al., 2020). For example, nitrogen deficiency in coconut plants leads to yellowing of mature leaves and a reduction in the number of female flowers, which ultimately hinders their normal growth and productivity (Broschat, 2009; Mohandas, 2012; Ramírez-Silva et al., 2021). Concurrently, coconut palms, predominantly distributed in tropical coastal regions, are susceptible to the challenges posed by drought and saline stress. Drought and salt stress initially leads to the death of leaf margins and tips, followed by wilting and yellowing (Wang et al., 2020; Samarasinghe et al., 2021; Yang et al., 2021). Interestingly, tall coconut varieties tend to exhibit greater drought tolerance compared to dwarf varieties (Sousa Santos et al., 2020), as well as the Hainan tall coconut, a local variety from Hainan Island, exhibits better salt tolerance than certain dwarf coconut varieties like aromatic coconut, yellow dwarf coconut and red dwarf coconut (Sun et al., 2024).

The NODULE-INCEPTION-Like Protein (NLP), a transcription factor unique to plants, is crucial for the transduction of nitrate signals and their assimilation, as well as for coping with abiotic stress and modulating plant growth and development (Konishi and Yanagisawa, 2014; Jia et al., 2023). In various plant species, including *Arabidopsis*, rice, apple, and tea, 9, 6, 6, and 33 *NLP* gene have been identified, respectively (Schauser et al., 2005; Wang et al., 2019; Jagadhesan et al., 2020; Li et al., 2024). The *NLP* gene mainly contains two conserved domains: the RWP-RK domain, which is responsible for DNA binding and specifically interacts with nitrate response *cis*-acting elements in the promoter region of the target genes (Chardin et al., 2014), and the PB1 domain, which is involved in nitrate-induced protein-protein interaction (Konishi and Yanagisawa, 2019). Additionally, a highly conserved GAF domain has been found at the N-terminal of some NLP proteins, which linked to signal transduction (Ho et al., 2000). The tissue-specific expression patterns of *NLP* genes in tomato, watermelon, and *Brassica napus* indicated that the expression patterns of *NLP* genes vary among different plant species. Specifically, *NLP* genes in tomato and watermelon are predominantly expressed in roots and leaves (Liu et al., 2021; Yuan et al., 2022), whereas in *Brassica napus*, *NLPs* are mainly expressed in leaves (Liu et al., 2018).

Functional studies have confirmed the key role of *NLP* genes in nitrogen response and assimilation processes. For instance, in *Arabidopsis*, *AtNLP* regulates primary nitrogen responses by binding to the nitrogen-responsive *cis*-acting elements (NRES) in the promoter region of target genes (Konishi and Yanagisawa, 2013). *AtNLP2* plays a critical role in nitrogen assimilation processes based on the availability of nitrate in the soil (Durand et al., 2023). In addition, *AtNLP7* interacts with key genes in the nitrogen metabolism pathway, such as *ANR1*, *NRT1.1*, *NRT2*, and *LBD37/38*, to regulate the expression of downstream genes (Marchive et al., 2013; Yu et al., 2016). *AtNLP8* has been demonstrated to directly bind to the promoter of abscisic acid catabolic enzyme genes, thereby reducing abscisic acid levels in a nitrate-dependent manner, and consequently regulating nitrate-promoted seed germination (Yan et al., 2016). Under conditions of persistent nitrate availabiliy and nitrogen starvation, *AtNLP6* and *AtNLP7* interact with another key transcriptional regulator *TCP20* and play an important role in regulating the expression of nitrate response genes (Guan et al., 2017; Kim et al., 2023). In rice, overexpression of *OsNLP1* has been associated with enhanced plant growth, increased grain yield, and improved nitrogen use efficiency, while the knockout of the *OsNLP1* gene under nitrogen restriction leads to a reduction in both grain yield and nitrogen utilization efficiency (Alfatih et al., 2020). Similarly, under conditions of nitrogen deficiency, the expression of *SlNLP1/2/4/6* genes in tomato was up-regulated, underscoring their important role in plant’s response to nitrogen limitation (Liu et al., 2021).

Notably, *NLPs* gene are involved in plant response to abiotic stress. For example, *AtNLP7* mutants are more tolerant to drought and salt than wild-type plants (Castaings et al., 2009; Le et al., 2022). Elephant grass and alfalfa studies have demonstrated that *NLP* genes respond to abiotic stresses (Jin et al., 2023; Yu et al., 2023). Specifically, *CpNLP5.2* in elephant grass exhibits rapid induction under drought and salt stress conditions (Jin et al., 2023). Similarly, in alfalfa, the expression of *MsNLP24* gene increases over time in response to drought and salt stress. In contrast, *MsNLP48* gene expression initially decreases, subsequently increases, but remains lower than the initial level, with the lowest expression observed at 6 h post-treatment (Yu et al., 2023). This suggests that salt and drought stress significantly induced or inhibited the expression of *MsNLPs* genes.

Despite the advances in understanding the role of NLP transcription factors in various biochemical and physiological processes in plants, there remains a significant gap regarding the function of NLP transcription factors in coconut. Comprehensive identification and systematic analysis of *NLP* gene family in coconut have yet to be conducted. Therefore, this study aims to systematically investigate the evolutionary characteristics, tissue-specific expression patterns, and responses to nitrogen deficiency, drought, and salt stress of *NLP* gene family members at the genome-wide level using the genomes of *Aromatic Dwarf* and *Hainan Tall* coconut. Additionally, we will explore the evolutionary relationship between dwarf coconut and tall coconut to identify the key genes involved in stress responses. Our goal is to provide insights into the evolutionary history and potential functional diversity of *NLP* gene in coconut.

## 2 Materials and methods

### 2.1 Identification and analysis of *CnNLP* family

Genome-wide data and gene structure annotation files of *Aromatic Dwarf* and *Hainan Tall* coconut varieties were downloaded from the China National Center for Bioinformation (https://ngdc.cncb.ac.cn/gwh/; accessed on 19 September 2023) under accession numbers GWHBEBT00000000 and GWHBEBU00000000, respectively (Wang et al., 2021). Protein sequences of *NLP* gene family members in *Arabidopsis thaliana* were sourced from The Arabidopsis Information Resource Database (https://www.arabidopsis.org/; accessed on 19 September 2023), and the candidate NLP proteins in coconut was identified using BLAST search. Furthermore, Hidden Markov Models (HMMs) for the RWP-RK (PF02042) and PB1 (PF00564) conserved domain of NLP protein were downloaded from the Pfam Database (https://www.ebi.ac.uk/interpro/entry/pfam/; accessed on 19 September 2023). The HMM search was performed with an E value threshold set at 1×10^-5^ to identify candidate NLP proteins in the coconut genome. To ensure accuracy, all candidate protein sequences obtained by the two methods were further verified using the NCBI Batch CD-search (https://www.ncbi.nlm.nih.gov/Structure/bwrpsb/bwrpsb.cgi; accessed on 19 September 2023).

The physicochemical properties of *NLP* gene family members in coconut, including the length of amino acids, molecular weight, isoelectric point, instability index, and grand average of hydropathicity (GRAVY), were analyzed using the ExPASy online tool (http://web.expasy.org/protparam/; accessed on 19 September 2023). The subcellular localization of these genes was predicted with the Plant-mPLoc online tool (http://www.csbio.sjtu.edu.cn/bioinf/plant-multi/; accessed on 19 September 2023). Furthermore, the chromosome gene density and the chromosome location of *NLP* genes in coconut were visualized using TBtools-II v2.086 (Chen et al., 2023).

### 2.2 Prediction of CnNLP protein structure

The secondary structure of NLP protein was predicted using the SOPMA online tool (https://npsa.lyon.inserm.fr/cgi-bin/npsa_automat.pl?page=/NPSA/npsa_sopma.html; accessed on 12 October 2023). Additionally, the tertiary structure of NLP protein was predicted by SWISS-MODEL online tool (https://swissmodel.expasy.org/interactive; accessed on 12 October 2023).

### 2.3 Prediction of interaction network of CnNLP protein

The interactions among *NLP* family member proteins were predicted using the functional protein association network online database STRING (https://cn.string-db.org/; accessed on 12 October 2023), with the model species selected as *Arabidopsis*.

### 2.4 Phylogenetic analysis of *CnNLP*

Download the protein sequences of *NLP* gene family in rice from the Rice Genome Annotation Project Database (http://rice.uga.edu/; accessed on 4 October 2023). Additionally, protein sequences of the validated *NLP* gene family from *Elaeis guineensis*, *Phoenix dactylifera*, *Spirodela polyrhiza*, *Ananas comosus*, *Musa acuminate*, *Vitis vinifera*, *Pyunus persica*, *Theobroma cacao*, *Sorghum bicolor*, *Glycine max*, and *Populus trichocarpa* were obtained from the Plant Transcription Factor Database (https://planttfdb.gao-lab.org/; accessed on 4 October 2023). The evolutionary tree was constructed using Neighbour-Joining method with 1000 bootstrap replicates, employing MEGA11 software (Tamura et al., 2021). The FigTree v1.4.4 software (Andrew Rambaut, IEB, UoE, UK) was utilized to visualize and analyze the evolutionary relationships.

### 2.5 Structural characteristics and conservative motifs of *CnNLP*

The evolutionary tree was constructed by Neighbour-Joining method (1000 bootstrap replicates) in MEGA11 software. The conserved motifs of *NLP* gene family proteins in coconut were analyzed using the online MEME tool (https://meme-suite.org/meme/tools/meme; accessed on 5 October 2023) with the number of conserved motifs set to 10 and the other parameters left at their default values. The exon/intron information of *NLP* gene, including mRNA, coding sequence (CDS), and untranslated region (UTR), was extracted from the coconut genome structure file.

### 2.6 Collinearity analysis of *CnNLP*

The KaKs_Calculator 2.0 (Wang et al., 2010) was utilized to evaluate the nucleotide substitution parameters of duplication genes: non-synonymous substitution rate (*Ka*) and synonymous substitution rate (*Ks*), and their ratio (*Ka/Ks*) was calculated. Using the method of Yao et al (Yao et al., 2021), the gene duplication events within the *NLP* family in coconut and the collinearity analysis among *Arabidopsis thaliana*, *Oryza sativa*, *Ananas comosus*, *Elaeis guineensis*, and *Cocos nucifera* were evaluated respectively. After that, we analyzed the origin within the *CnNLP* gene family using BLAST comparison. The genome-wide data and gene structure annotation file of *Ananas comosus* were obtained from the Phytozome Database (https://phytozome-next.jgi.doe.gov/; accessed on 5 October 2023), and those for *Elaeis guineensis* were downloaded from the National Center for Biotechnology Information Database (https://www.ncbi.nlm.nih.gov/; accessed on 5 October 2023).

### 2.7 Analysis of *CnNLP* gene promoter

The 2000 bp sequence upstream of the gene transcription start site was extracted as a putative promoter region. Subsequently, the extracted sequence was submitted to PlantCare (http://bioinformatics.psb.ugent.be/webtools/plantcare/html/; accessed on 4 October 2023) for cis-acting element prediction (Wu et al., 2021; Rao et al., 2021).

### 2.8 Plant growth and treatments

The experiment was conducted in December 2023 at Sanya Breeding Institute of Hainan University (18° 30’ N, 109° 60’ E). Coconut seedlings cultivated for one year (*Cocos nucifera L.* cv. *Aromatic Dwarf* and *Cocos nucifera L.* cv. *Hainan Tall*) were selected as the experimental materials. Fresh samples of roots, petioles, young, and mature leaves from both *Aromatic Dwarf* and *Hainan Tall* seedlings were collected, with three replicates for each treatment, and then stored at -80°C. In addition, *Aromatic Dwarf* seedlings were incubated in clean water for 3 days before being transferred to an abiotic stress environment for further cultivation. The treatments included nitrogen deficiency stress using nitrogen-deficient Hoagland nutrient solution, salt stress with a normal Hoagland nutrient solution containing 300 mmol/L NaCl, and drought stress using a normal Hoagland nutrient solution with 25% PEG6000. Roots, petioles, young, and mature leaves were collected at 0, 3, 6, 12, 24, and 48 h post-treatment, respectively, and stored in the refrigerator at -80 °C after being preserved liquid nitrogen.

### 2.9 qRT-PCR analysis and homologous gene screening

Total RNA was extracted using an RNA extraction kit according to the manufacturer’s protocol (Tiangen, Beijing, China, DP437). RNA was reverse-transcribed into cDNA using HiScripv II Q RT SuperMix for quantitative real-time PCR (qRT-PCR) (Vazyme, Nanjing, China, R223). The SYBR Green I chimeric fluorescence method was employed for quantitative qRT-PCR reaction on qTOWER3G Real-Time PCR Thermal Cycler (Analytik Jena, Germany). The *β-actin* gene of coconut was selected as the internal control. The relative expression was calculated by 2^−ΔΔCT^method. Three biological replicates were set up for each sample. The primers used in this experiment are listed in **Supplementary Table S1**. Homologous genes of *NLP* in coconut were screened using the Quick Find Best Homology functions in TBtools (Chen et al., 2023). Building an evolutionary tree using MEGA11 software (Tamura et al., 2021).

### 2.10 Statistical analysis

All data were statistically analyzed using Microsoft Excel 2019 (Microsoft, Redmond, WA, USA). The analysis software Statistix 9 (Analytical Software, Tallahassee, FL, USA) was used to conduct one-way ANOVA. Graphs in this study were visualized using GraphPad Prism 8.0.2.263 (GraphPad Software Inc., San Diego, CA, USA), Origin 2024 (OriginLab Corporation, Northampton, MA, USA), and TBtools-II v2.086 (Chen et al., 2023).

## 3 Results

### 3.1 Identification and basic characteristics of CnNLP transcription factor family

**Figure 1:**
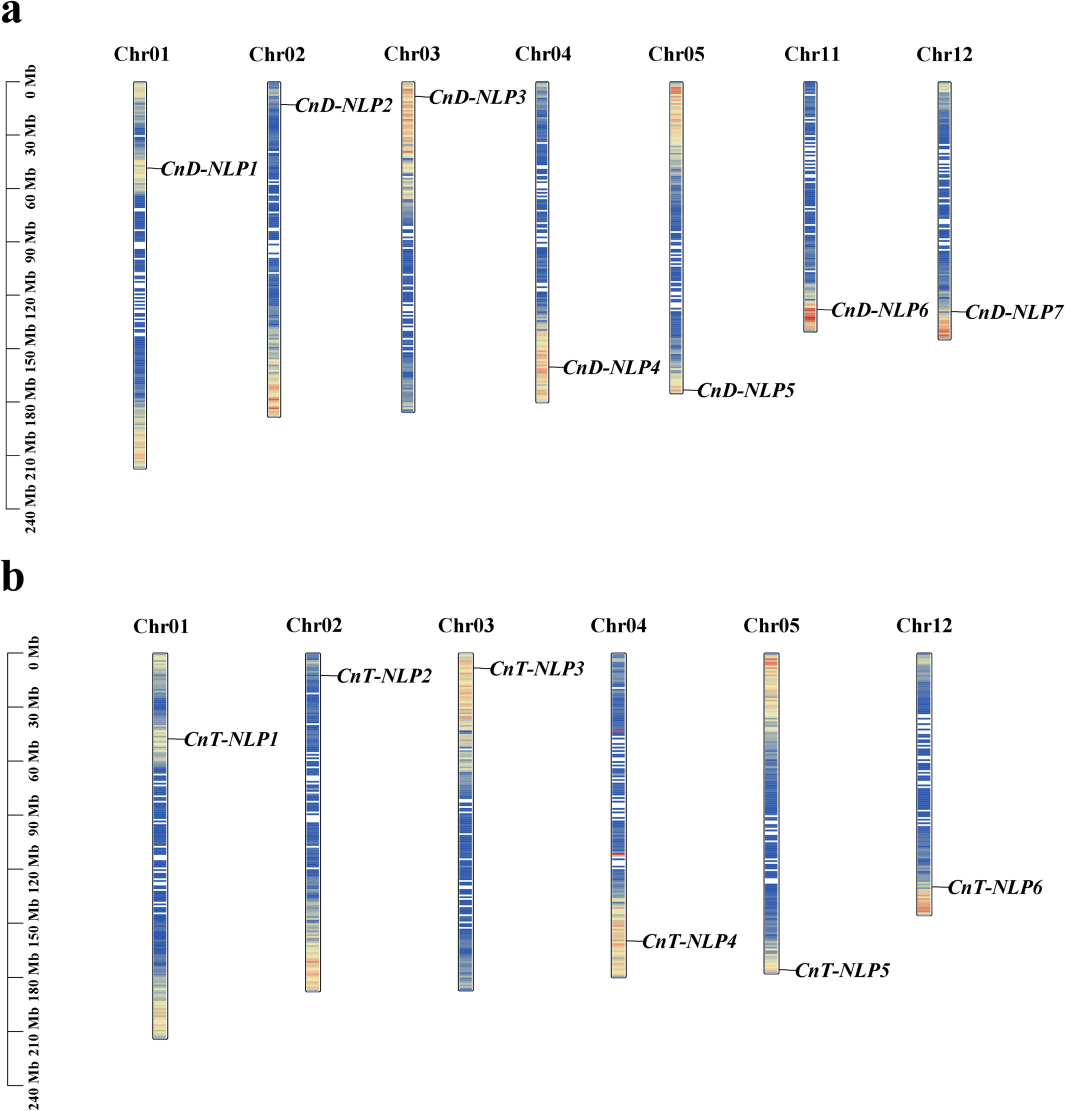
Chromosome location and distribution of *NLP* gene in *Aromatic Dwarf* **(a)** and *Hainan Tall* **(b)**. These bars represent chromosomes. The chromosome number is displayed at the top and the gene name is displayed on the right. The relative chromosomal location of each *NLP* gene is located on the black line on the right. Blue to red on the chromosome indicates gene density.

After performing BLAST sequence alignment, HMM search, redundancy removal, and protein domain screening, 7 *NLP* genes were identified in *Aromatic Dwarf* and 6 *NLP* genes in *Hainan Tall*. Based on their chromosomal positions (**Figure 1**), the 7 *NLP* genes in *Aromatic Dwarf* were designated as *CnD-NLP1* to *CnD-NLP7*, while the 6 *NLP* genes in *Hainan Tall* were designated as *CnT-NLP1* to *CnT-NLP6*. These genes were distributed across different chromosomes, with most of them located near the chromosome ends (**Figure 1**). Protein characterization analysis revealed that the *CnD-NLP* gene family members have amino acid lengths ranging from 734 (*CnD-NLP1*) to 959 (*CnD-NLP7*) and relative molecular weights between 81.54 kDa (*CnD-NLP1*) and 106.49 kDa (*CnD-NLP2*). The isoelectric points of these proteins range from 5.44 (*CnD-NLP4*) to 6.56 (*CnD-NLP1*), and their instability indices range from 49.55 (*CnD-NLP6*) to 55.57 (*CnD-NLP2*). The grand average of hydropathicity (GRAVY) values for these proteins range from -0.447 (*CnD-NLP6*) to -0.317 (*CnD-NLP3*). Similarly, the *CnT-NLP* gene family encodes proteins with amino acid lengths from 734 (*CnT-NLP1*) to 959 (*CnT-NLP6*) and relative molecular weights between 81.54 kDa (*CnT-NLP1*) and 106.59 kDa (*CnT-NLP2*). Their isoelectric points range from 5.44 (*CnT-NLP4*) to 6.56 (*CnT-NLP1*), and their instability indices range from 51.68 (*CnT-NLP4*) to 55.88 (*CnT-NLP2*), with GRAVY values ranging from -0.442 (*CnT-NLP1*) to -0.317 (*CnT-NLP3*). Both *CnD-NLP* and *CnT-NLP* gene family members are characterized as acidic proteins with isoelectric points below 7, unstable proteins with instability indices above 40, and hydrophilic proteins with negative GRAVY values. According to Plant-mPLoc prediction, all *CnD-NLPs* and *CnT-NLPs* proteins are localized in the nucleus (**Table 1**).

**Table 1:** Characteristics of NLP family members in coconut.

Gene name	Gene ID	Chromosome location	+/–	From	To	Protein length (aa)	MW (kDa)	PI	Instability Index	GRAVY	Subcellular localization
*CnD-NLP1*	AZ01G0008550.1	Chr01	+	48,621,087	48,628,027	734	81.54	6.56	54.62	-0.442	Nucleus
*CnD-NLP2*	AZ02G0027780.1	Chr02	+	12,892,579	12,932,403	956	106.49	5.79	55.57	-0.419	Nucleus
*CnD-NLP3*	AZ03G0050330.1	Chr03	+	8,393,995	8,399,977	953	104.34	5.52	52.81	-0.317	Nucleus
*CnD-NLP4*	AZ04G0089750.1	Chr04	–	160,314,098	160,320,023	957	105.14	5.44	51.68	-0.332	Nucleus
*CnD-NLP5*	AZ05G0122270.1	Chr05	–	173,171,218	173,176,146	895	98.51	6.29	53.21	-0.339	Nucleus
*CnD-NLP6*	AZ11G0215320.1	Chr11	+	128,021,224	128,028,148	920	101.91	5.93	49.55	-0.447	Nucleus
*CnD-NLP7*	AZ12G0228810.1	Chr12	+	129,262,254	129,274,746	959	105.00	5.87	51.73	-0.369	Nucleus
*CnT-NLP1*	GZ01G0008960.1	Chr01	+	47,612,758	47,619,738	734	81.54	6.56	54.62	-0.442	Nucleus
*CnT-NLP2*	GZ02G0028770.1	Chr02	+	12,529,996	12,569,682	956	106.59	5.63	55.88	-0.423	Nucleus
*CnT-NLP3*	GZ03G0052860.1	Chr03	+	8,447,097	8,452,944	953	104.34	5.52	52.81	-0.317	Nucleus
*CnT-NLP4*	GZ04G0096300.1	Chr04	–	159,709,655	159,715,670	957	105.14	5.44	51.68	-0.332	Nucleus
*CnT-NLP5*	GZ05G0131290.1	Chr05	–	175,796,144	175,801,458	895	98.51	6.29	53.21	-0.339	Nucleus
*CnT-NLP6*	GZ12G0242730.1	Chr12	+	129,701,168	129,707,348	959	105.00	5.87	51.73	-0.369	Nucleus

MW, molecular weight; pI, isoelectric point; GRAVY, grand average of hydropathicity. (+) means the forward orientation of the gene in the corresponded chromosome; (–) means the reverse orientation of the gene in the corresponded chromosome.

### 3.2 Structure and interaction network analysis of CnNLP protein

**Figure 2:**
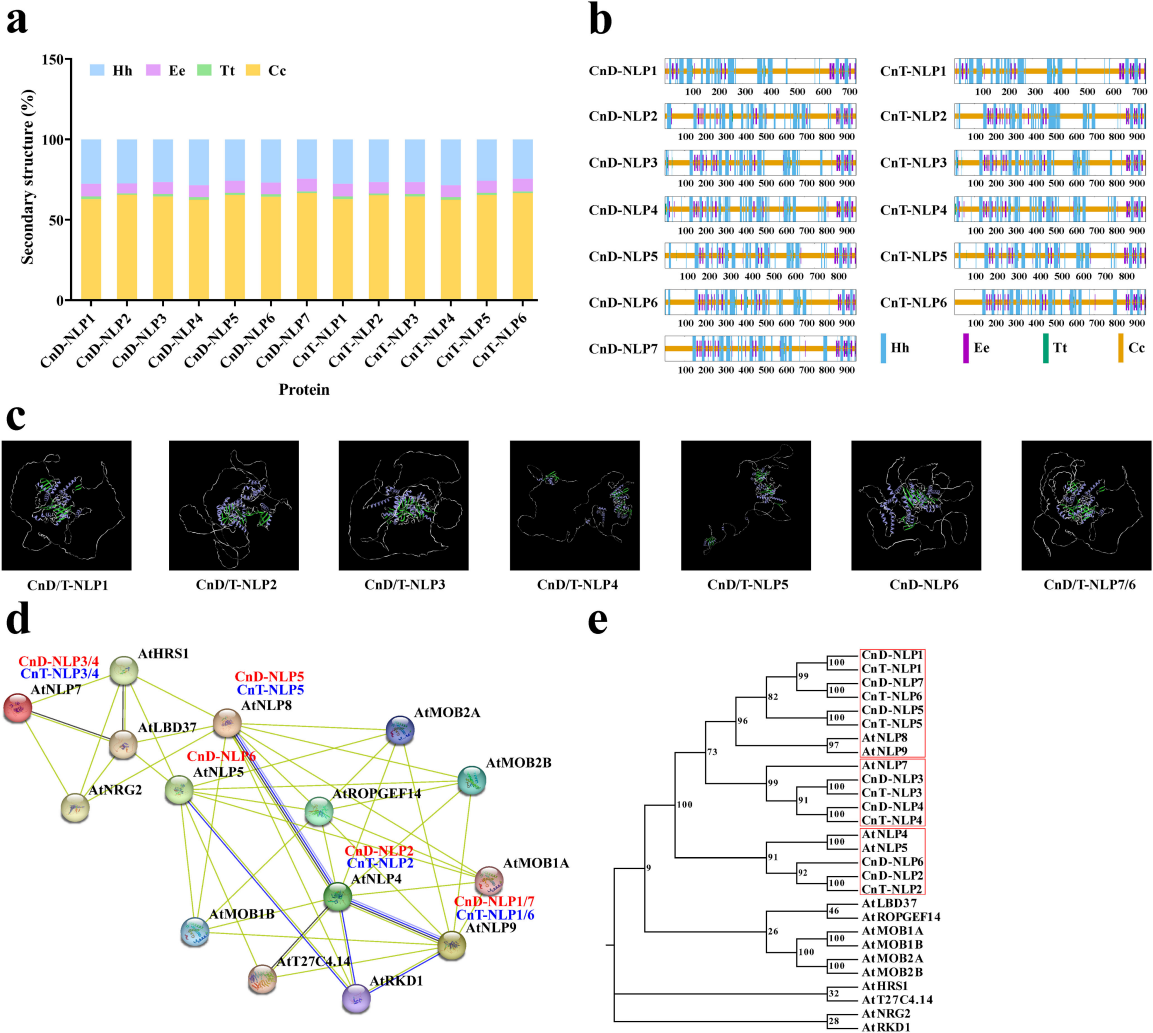
Protein structure and protein interaction network of NLP in coconut. **(a, b)** Proportion and distribution of secondary structure element. Hh: Alpha helix; Ee: Extended strand; Tt: Beta turn; Cc: Random coil. **(c)** The predicted tertiary structure of NLP protein in *Aromatic Dwarf* and *Hainan Tall*. Purple represents the Alpha helix. Green represents the β-sheet. Gray represents the Random coil. **(d)** Protein interaction network of NLP in *Aromatic Dwarf* and *Hainan Tall* constructed by referring to AtNLPs. The different data sources of predicted interactions are indicated by lines with diverse colors. Dark blue represents gene co-occurrence. Chartreuse represents text mining. Black represents co-expression. Light blue represents protein homolog. **(e)** Phylogenetic analysis of NLP proteins from coconut, *Arabidopsis*, and their interacting proteins.

The secondary structure prediction of CnNLP proteins revealed that the 13 NLP proteins were mainly consisted of random coil and α-helix, with random coils being the most prevalent, ranging between 62.49% to 66.63%. In addition, the secondary structure included extended strands and β-turn, with β-turn representing the smallest proportion among all NLP members, ranging from 0.84% to 1.77%. Notabaly, the proportion of these secondary structures were largely consistent across the proteins (**Figures 2a, b**; **Supplementary Table S2**). Using SWISS-MODEL, we predicted the tertiary structures of these proteins. The GMQE values for the 13 models ranged from 0.59 to 0.62, with sequence similarities exceeding 70% (**Supplementary Table S3**), indicating that the predicted tertiary structures are reliable. The structural analysis showed that there are certain differences in the tertiary structure among proteins within the same variety, suggesting potential diversity among the members of the *NLP* gene family. Furthermore, we found that CnD-NLP1 and CnT-NLP1, CnD-NLP2 and CnT-NLP2, CnD-NLP3 and CnT-NLP3, CnD-NLP4 and CnT-NLP4, CnD-NLP5 and CnT-NLP5, CnD-NLP7 and CnT-NLP6 are homologous genes with highly similar sequences and the same predicted tertiary structure (**Figure 2c**), indicating that these may have similar functions.

To predict the potential function of *NLP* gene in coconut, we constructed an interaction network of coconut NLP proteins using the homologous sequences of the model species *Arabidopsis thaliana*, employing the STRING database (**Figure 2d**). Subsequently, we constructed a phylogenetic tree that includes the NLP proteins of *Arabidopsis* and their interacting proteins, as well as the NLP protein sequences from coconut (**Figure 2e**). In this network, CnD-NLP3/4 and CnT-NLP3/4 corresponds to AtNLP7, CnD-NLP5 and CnT-NLP5 corresponds to the AtNLP8 (**Figures 2d, e**; **Supplementary Table S4**). The network diagram illustrates that the AtNLP7/8 protein can interact with the nitrate-regulating gene AtNRG2, as well as transcription factors AtHRS1 and AtLBD37, among others. Based on these interactions, we predict that CnD-NLP3/4/5 and CnT-NLP3/4/5 play key roles in nitrogen utilization in coconut.

### 3.3 Phylogenetic and structural analysis of *NLP* gene

**Figure 3:**
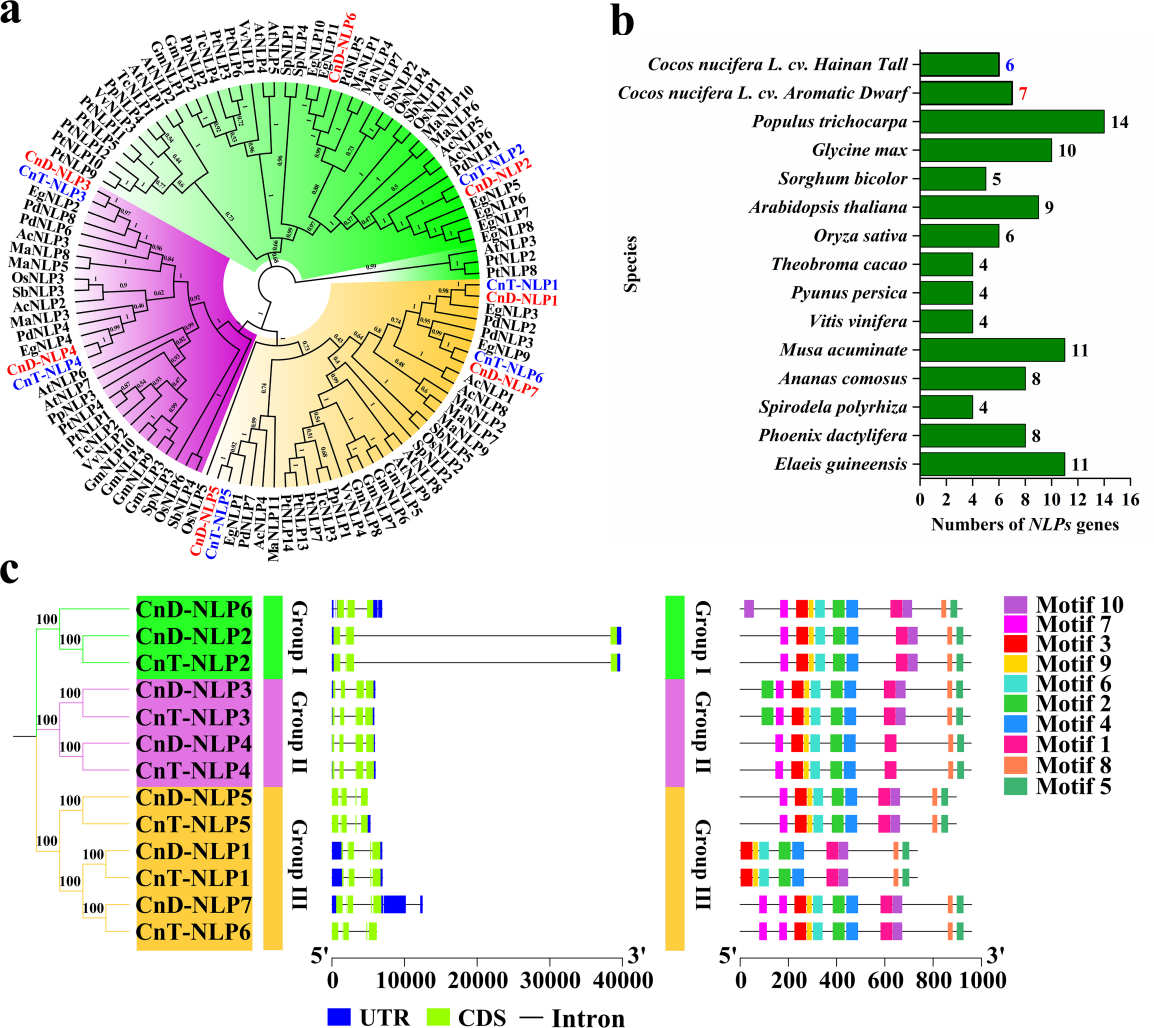
Phylogenetic and structural analysis of *NLP* genes. **(a)** Phylogenetic analysis of the NLP proteins from different species. The phylogenetic tree was divided into three groups, each with a different colour. The values in the branch represent bootstrap values. **(b)** Statistics of *NLP* gene numbers across 15 plant species. **(c)** Phylogenetic tree, gene structure and conserved motif of *NLP* gene family in coconut. UTR, untranslated region; CDS, coding sequence.

To evaluate the evolutionary relationship among plant *NLP* genes, we constructed a phylogenetic tree using 111 NLP amino acid sequences from 15 angiosperms species encompassing both monocots and dicots (**Figure 3a**; **Supplementary Table S5**). Following the classification from *Arabidopsis thaliana* (Schauser et al., 2005), the NLP family was divided into three groups: Group I (in green) includes CnD-NLP2/6 and CnT-NLP2; Group II (in purple) includes CnD-NLP3/4 and CnT-NLP3/4; and Group III (in orange) includes CnD-NLP1/5/7 and CnT-NLP1/5/6. Furthermore, the dendrogram also revealed closely related orthologs among *Cocos nucifera L.* cv. *Aromatic Dwarf*, *Cocos nucifera L.* cv. *Hainan Tall*, *Elaeis guineensis*, and *Phoenix dactylifera* (for instance, CnD-NLP1/CnT-NLP1/EgNLP3/PdNLP2), suggesting that these genes may share similar functions. To further understand the evolution of *NLP* genes, we counted the number of *NLP* genes in 15 herbaceous and woody plants (**Figure 3b**). The number of *NLP* genes varied among the plants, with *Populus trichocarpa* having the highest number (14), while *Spirodela polyrhiza* (4), *Vitis vinifera* (4), *Pyunus persica* (4), and *Theobroma cacao* (4) each having the lowest numbers.

The exon-intron distribution of the *CnNLP* genes was analyzed, revealing minor variations in the number of exons and introns among different genes The *CnNLP* genes contained four to five exons and three to six introns. Notably, Group II and Group III of *CnNLP* genes each contained five exons, whereas Group I of *CnNLP* gene contained four exons. Additionally, the number of *CnNLP* exons within the same group was consistent across the genes (**Figure 3c**). Furthermore, the sequence motifs in *CnNLP* were analyzed using the MEME website, and 10 motifs were identified, named as Motif1-Motif10 (**Figure 3c**; **Supplementary Figure S1**). Most genes contained all 10 conserved motifs, except for *CnD-NLP4* and *CnT-NLP4* (which lacked Motif10 and thus had only 9 conserved motifs), *CnD-NLP1* and *CnT-NLP1* (which lacked Motif7 and also had only 9 conserved motifs).

### 3.4 Collinear analysis of *CnNLP*

**Figure 4:**
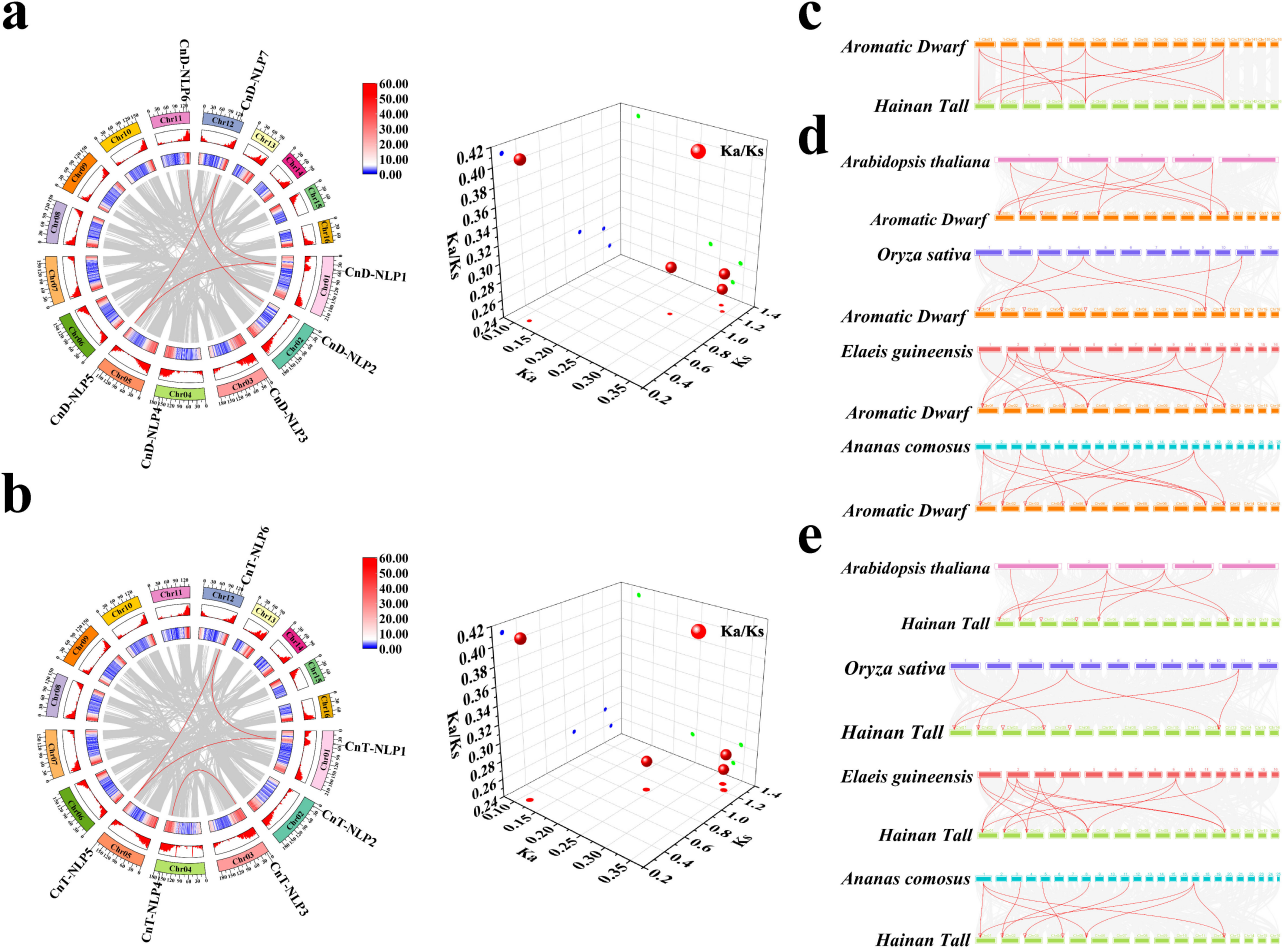
Gene collinearity analysis. **(a, b)** Collinear analysis and *Ka/Ks* analysis of 3D scatter plots of *NLP* gene in *Aromatic Dwarf* and *Hainan Tall*. The gray lines in the background represent collinear blocks in the coconut genome. The red lines indicate that the repeated *NLP* gene pairs of coconut are collinear. The number of chromosomes is shown inside each chromosome. The bars and heat maps of the outer circle represent the gene density on the chromosome. **(c–e)** Homology analysis of *NLP* gene between coconut and other representative plants. The gray lines in the background represent collinear blocks in the genomes of coconuts and other plants. The red lines represent the regions where the *CnNLP* gene is significantly collinear between plant genomes. The red triangle represents the position of the *CnNLP* gene on the coconut chromosome.

Collinearity analysis found that seven members of the *CnD-NLP* gene family have four repetitive gene pairs (**Figure 4a**), and six members of the *CnT-NL*P gene family have four repetitive gene pairs (**Figure 4b**). To further explore the evolutionary origins of the *CnNLP* gene family, we selected chromosomal fragments containing the *CnNLP* gene to identify regions of collinearity. Our analysis revealed that the fragment containing *CnD-NLP1* on chromosome 01 exhibited high collinearity with the fragment containing *CnD-NLP7* on chromosome 12. Similarly, the fragment containing *CnD-NLP2* on chromosome 02 was found to be highly collinear with the fragment containing *CnD-NLP6* on chromosome 11, and the fragment containing *CnD-NLP3* on chromosome 03 was highly collinear with the fragment containing *CnD-NLP4* on chromosome 04. Notably, the fragment containing *CnD-NLP5* on chromosome 05 was not identified (**Supplementary Figure S2a**). Subsequently, we compared the chromosomal fragment containing the *CnNLP* gene with that containing the *AcNLP* gene. This comparison demonstrated that the fragment containing *CnD-NLP1* on chromosome 01 was highly collinear with the fragment containing *AcNLP1* on chromosome 17. The fragment containing *CnD-NLP2* on chromosome 02 was highly collinear with the fragment containing *AcNLP6* on chromosome 03, and the fragment containing *CnD-NLP3* on chromosome 03 was highly collinear with the fragment containing *AcNLP3* on chromosome 11. Additionally, the fragment containing *CnD-NLP5* on chromosome 05 was found to be highly collinear with the fragment containing *AcNLP4* on chromosome 01 (**Supplementary Figure S2b**). Therefore, we conclude that *CnD-NLP5* likely originated prior to species differentiation, while other members of the *CnD-NLP* gene family may have originated from whole-genome duplication (WGD) events. Similarly, the *CnT-NLP* gene family also showed similar patterns of collinearity, leading us to inferr that *CnT-NLP2* and *CnT-NLP5* may have originated before species differentiation, while other members of *CnT-NLP* gene family potentially originated from WGD events (**Supplementary Figures S2c, d**). Further analysis of the selection types for these repetitive gene pairs whthin the *CnD-NLP* and *CnT-NLP* gene families revealed that the *Ka/Ks* values of all repetitive gene pairs were less than 1, indicating that the expansion of these gene families was mainly affected by purifying selection (**Figures 4a, b**; **Supplementary Table S6**).

To further investigate the evolutionary relationship of *NLP* genes across different plant species, including *Arabidopsis thaliana*, *Oryza sativa*, *Elaeis guineensis*, *Ananas comosus*, and *Cocos nucifera*, a collinear map was constructed. The results showed that 7 *CnD-NLP* genes and 6 *CnT-NLP* genes formed homologous pairs with 14 collinear relationships (**Figure 4c**). Furthermore, 5 *CnD-NLP* genes and 5 *AtNLP* genes formed homologous pairs with 12 collinear relationships, 5 *CnD-NLP* genes and 5 *OsNLP* genes formed homologous pairs with 8 collinear relationships, 7 *CnD-NLP* genes and 7 *EgNLP* genes formed homologous pairs with 15 collinear relationships, 7 *CnD-NLP* genes and 7 *AcNLP* genes formed homologous pairs with 13 collinear relationships (**Figure 4d**). Similarly, 4 *CnT-NLP* genes and 5 *AtNLP* genes formed homologous pairs with 9 collinear relationships, 4 *CnT-NLP* genes and 4 *OsNLP* genes formed homologous pairs with 6 collinear relationships, 6 *CnT-NLP* genes and 7 *EgNLP* genes formed homologous pairs with 15 collinear relationships, and 6 *CnT-NLP* genes and 6 *AcNLP* genes formed homologous pairs with 10 collinear relationships (**Figure 4e**). These findings suggest that the *NLP* gene family in coconut is closely related to the *NLP* gene family in oil palm, indicating that these genes may share similar functions.

### 3.5 Analysis of *CnNLP* gene *cis*-regulatory elements

To further explore the potential function of the *CnNLP* genes, the upstream 2000 bp sequence was extracted as the promoter region and analyzed using PlantCare to predict *cis*-acting elements. Various *cis*-acting elements were identified, including light responsive, hormone responsive, stress responsive, and growth and development response elements. In the *CnNLP* gene family, *CnD-NLP6/7* and *CnT-NLP6* contained defense-related stress elements (TC-rich repeats), while *CnD-NLP1/4/7* and *CnT-NLP1/4/6* contained multiple drought response elements (MBS), suggesting that these genes may be involved in the coconut’s stress response (**Supplementary Figure S3**).

### 3.6 Effects of abiotic stress on coconut seedlings growth

**Figure 5:**
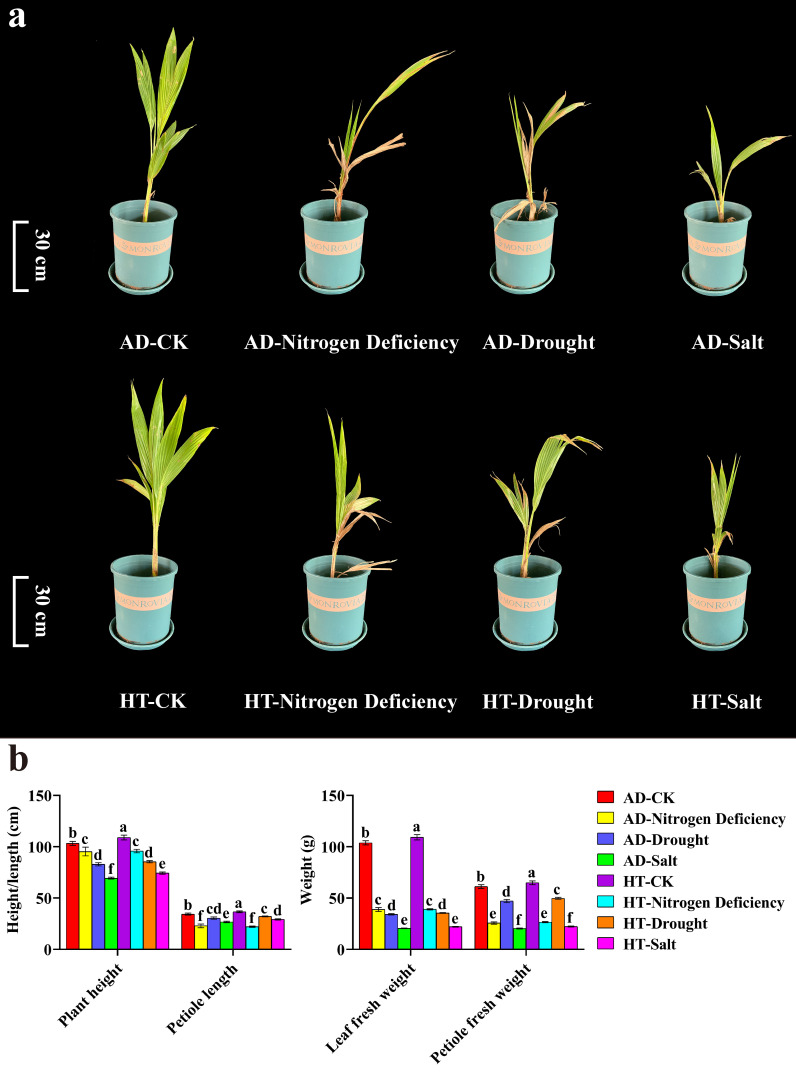
Growth and development of coconut under different varieties and treatments. AD stands for *Aromatic Dwarf*. HT stands for *Hainan Tall*. **(a)** Phenotypic changes of Coconut under nitrogen deficiency, drought, and salt stress. **(b)** The plant height, petiole length, leaf fresh weight, and petiole fresh weight under CK, nitrogen deficiency, drought, and salt treatments. In the one-way ANOVA, the values represented by different lowercase letters were significantly different at *p* < 0.05.

To understand the impact of abiotic stress on the growth of different coconut varieties, *Aromatic Dwarf* and *Hainan Tall* were subjected to nitrogen deficiency, drought stress, and salt stress. Compared to the control, the plant height, petiole length, leaf fresh weight, and petiole fresh weight of both varieties of decreased significantly (**Figure 5b**). Under nitrogen deficiency stress, the lower leaves of both varieties withered. Under drought stress, the leaves of *Aromatic Dwarf* wilted extensively, while only the tips of the upper and lower leaves of the *Hainan Tall* wilted. Under salt stress, some leaves of both coconut varieties turned yellow and dry (**Figure 5a**). Together, *Hainan Tall* showed strong tolerance to different abiotic stresses, leading to the selection of *Aromatic Dwarf* as the further research object.

### 3.7 Gene expression profiles of CnNLP transcription factor family and key gene screening

**Figure 6:**
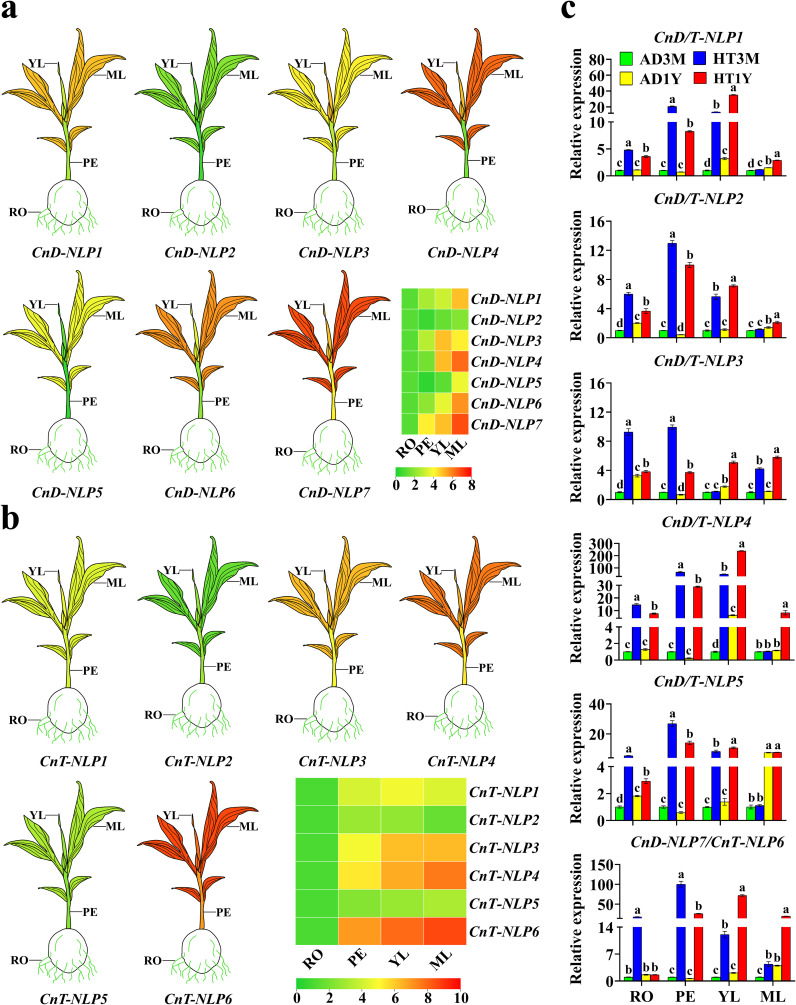
Gene expression analysis of *NLP* gene in coconut. AD stands for *Aromatic Dwarf*, HT stands for *Hainan Tall*; 3M represents coconut cultivated for three months, 1Y represents coconut cultivated for one year; RO, roots; PE, petioles; YL, young leaves; MF, mature leaves. **(a, b)** Differential expression of representative *CnNLP* genes in different tissues. The mean expression value was calculated from three independent biological replicates and is expressed in relate to that in the roots. Data is converted by Log to generate the heatmap. The red represents high expression levels, and the green represents low expression levels. **(c)** Analysis of *NLP* gene expression in different growth stages of coconut. The mean expression values were calculated from three independent biological replicates and are relative to those of the AD3M controls. In the one-way ANOVA, the values represented by different lowercase letters were significantly different at *p* < 0.05.

Through bioinformatics analysis, it was found that *CnD-NLP1* and *CnT-NLP1*, *CnD-NLP2* and *CnT-NLP2*, *CnD-NLP3* and *CnT-NLP3*, *CnD-NLP4* and *CnT-NLP4*, *CnD-NLP5* and *CnT-NLP5*, *CnD-NLP7* and *CnT-NLP6* were homologous genes. The expression patterns of *NLP* genes in *Aromatic Dwarf* and *Hainan Tall* were studied using qRT-PCR across tissues, including roots, petioles, young leaves, and mature leaves (**Figures 6a, b**). These genes exhibited tissue-specific transcriptional accumulation patterns. Notably, the expression profiles of *CnD-NLP2* and *CnT-NLP2/5* were similar across different tissues. The expression levels of other genes were higher in leaves (including young leaves and mature leaves) compared to in roots and petioles. *CnD-NLP3* and *CnT-NLP1* were highly expressed in young leaves, whereas *CnD-NLP1/4/5/6/7* and *CnT-NLP3/4/6* were highly expressed in mature leaves. The specific expression of *CnNLP* genes in leaves suggested their involvement in leaf growth and development.

**Figure 7:**
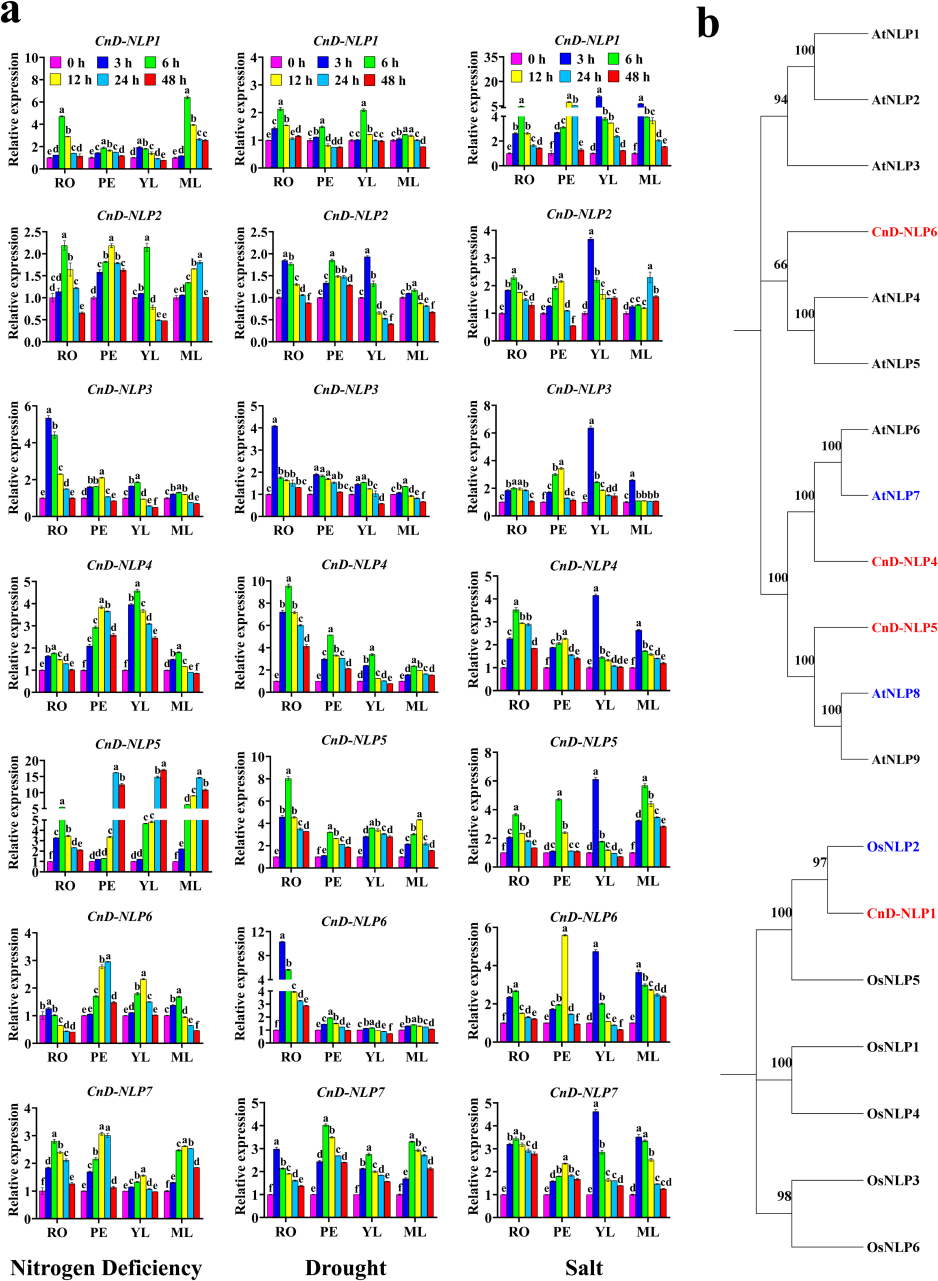
Key gene analysis. **(a)** Expression profiles of *CnD-NLP* genes in response to different stress treatments. RO, roots; PE, petioles; YL, young leaves; MF, mature leaves. The mean expression values were calculated from three independent biological replicates and are relative to those of the 0 h controls. In the one-way ANOVA, the values represented by different lowercase letters were significantly different at *p* < 0.05. **(b)** Screening of homologous genes of key genes. The red mark represents key genes in the *CnNLP* gene family that may respond to nitrogen deficiency, drought and salt stress, and the blue mark represents *Arabidopsis* and rice *NLP* genes with similar functions. *AtNLP8* is a nitrogen-related gene, *AtNLP7* is a drought-resistant gene, *OsNLP2* responds to salt stress.

To further understand the role of *NLP* genes in coconut development, the qRT-PCR technique was employed to analyze the expression of *CnD-NLPs* and *CnT-NLPs* at two growth stages. The results indicated that, at the same growth stage, *NLP* gene expression was higher in *Hainan Tall’s* tissues than in *Aromatic Dwarf’s*, indicating that *Hainan Tall* possessed stronger stress resistance. In *Aromatic Dwarf* seedlings cultured for 3 months and 1 year, *NLP* genes expression in AD3M petioles was higher than in AD1Y, while the expression in AD3M roots, young leaves, and mature leaves was lower than in AD1Y. Consequently, *Aromatic Dwarf* seedlings cultured for 1 year were selected for abiotic stress study (**Figure 6c**). The results showed that the expression of 7 *CnD-NLP* genes varied across tissues with the passage of time under abiotic stress (**Figure 7a**). Notably, under nitrogen deficiency, drought, and salt stress, the expression of *CnD-NLP5* gene in young leaves increased continuously under nitrogen deficiency stress, while the expression of other genes in different tissues increased at first and then decreased, and reached the peak at a specific time point. This shows that the response of *NLP* to nitrogen deficiency, drought, and salt stress is mainly within 24 hours, and the response is the most intense in 3-6 hours. Under nitrogen deficiency stress, the expression of *CnD-NLP5* in petioles, young leaves, and mature leaves changed significantly. After 24 hours of stress, *CnD-NLP5* expression in petioles and mature leaves reached the maximum level (16.2 and 14.7 times of the expression level at 0 h, respectively), and after 48 hours, the expression of *CnD-NLP5* in young leaves reached the maximum level (17.1 times of the expression level at 0 h). It is speculated that *CnD-NLP5* may be the key gene in its response to nitrogen deficiency stress. Under drought stress, the expression of *CnD-NLP4/5/6* genes in roots changed significantly. After 3 hours, *CnD-NLP6* reached its maximum level in roots (10.3 times of the expression level at 0 h), and after 6 hours, the expression of *CnD-NLP4/5* in roots reached the maximum level (9.6 and 8.0 times of the expression level at 0 h, respectively). Therefore, these three genes may play a key role in response to drought stress. Under salt stress, *CnD-NLP1* gene expression in petioles, young leaves, and mature leaves showed significant changes. After 3 hours, the expression of *CnD-NLP1* reached its peak in young leaves and mature leaves, which were 10.8 times and 6.4 times higher than that at 0 h, respectively. In petioles, the expression level of *CnD-NLP1* reached its highest level 12 h after stress, which was 7.4 times higher than that at 0 h. This suggests that *CnD-NLP1* may be a key gene in response to salt stress. In addition, we found that the expression of all genes in young leaves peaked after 3 hours of salt stress and then gradually decreased, indicating that salt stress can quickly induce the expression of *NLP* genes in coconut young leaves.

Homology screening of the key genes revealed that *CnD-NLP5* shared homology with *AT2G43500.5* (*AtNLP8*) (**Figure 7b**). Previous studies have shown that *AtNLP8* reduces abscisic acid levels in a nitrate-dependent manner to regulate nitrate-promoted seed germination (Yan et al., 2016), indicating that *CnD-NLP5* may serve a similar function. Additionally, *CnD-NLP4* has a high degree of homology with *AT4G24020.1* (*AtNLP7*) after strict screening (**Figure 7b**). It is known that *AtNLP7* mutants exhibit greater drought tolerance compared to wild-type plants (Castaings et al., 2009), indicating that *CnD-NLP4* may play a key role in responding to drought stress. Furthermore, the homologous gene *LOC_Os04g41850.1* (*OsNLP2*) of *CnD-NLP1* was identified in the rice genome (**Figure 7b**). Previous studies found that salt stress induced *OsNLP2*, which upregulated the expression of *OsNR*, thereby promoting salt tolerance during seed germination (Yi et al., 2022). This indicates that *OsNLP2* responds to salt stress, suggesting that *CnD-NLP1* may a similar function. These results further demonstrate the role of *CnD-NLP5*, *CnD-NLP4*, and *CnD-NLP1* in coconut’s responses to nitrogen deficiency, drought stress, and salt stress.

## 4 Discussion

In this study, 7 *CnD-NLP* genes and 6 *CnT-NLP* genes were identified in *Aromatic Dwarf* and *Hainan Tall* varieties, respectively (**Table 1**). Previous studies have revealed that there are 9 *NLP* genes in *Arabidopsis* and maize (Schauser et al., 2005; Wang et al., 2018), 6 *NLP* genes in rice and apple (Wang et al., 2019; Jagadhesan et al., 2020), which is comparable to the members of *NLP* genes in coconut. However, *NLP* gene numbers vary significantly among species, including 53 in alfalfa, 33 in tea, and 15 in elephant grass (Jin et al., 2023; Yu et al., 2023; Li et al., 2024), indicating that species-specific variation in *NLP* genes numbers.

Interestingly, *NLP* family genes were found to encode longer proteins and have lower isoelectric points in many species (Schauser et al., 2005; Chardin et al., 2014; Cheng et al., 2014), and similar results were observed in *NLP* genes of coconut (**Table 1**). This suggests that this transcription factor may be more active under acidic conditions. Subcellular localization prediction showed that all *CnD-NLPs* and *CnT-NLPs* are located in the nucleus (**Table 1**), consistent with the characteristics of transcription factors. Conserved motif analysis of NLP members in two coconuts varieties revealed similar distributions of conserved motifs within the same group (**Figure 3c**), indicating that NLP members in the same group may have similar biological functions. Gene structure analysis revealed significant differences in the lengths of coconut *NLP* genes, with most varying from *CnD-NLP5* (4929 bp) to *CnD-NLP7* (12493 bp), and a few, such as *CnD-NLP2* (39825 bp) and *CnT-NLP2* (39687 bp), having longer sequences (**Table 1**; **Figure 3c**). Previous studies have shown that the acquisition or loss of exons or introns contributes to structural differences and functional diversity of genes (Xu et al., 2012). A comprehensive analysis of the sequence length and exon-intron structure of *NLP* genes in coconut revealed that introns, particularly those in *CnD-NLP2* and *CnT-NLP2*, significantly affect sequence length, indicating their importance in exon splicing regulation. Phylogenetic analysis showed that coconut NLPs were divided into three groups. Coconut, oil palm, and date palm NLPs were clustered in close branches in each group, reflecting their close relationship as members of Palmaceae (**Figure 3a**). Gene duplication events play a crucial role in the development of the genome, allowing new functions and evolutionary pathways to be acquired (Qiao et al., 2018, 2019). This study suggests that *CnD-NLP5*, *CnT-NLP2* and *CnT-NLP5* likely originated prior to species differentiation, while other members of the *CnD-NLP* and *CnT-NLP* gene family may have originated from the WGD event. We speculate that the amplification of the *CnNLP* gene family may have been driven by WGD (**Supplementary Figure S2**).

Tissue-specific expression of *NLP* genes has been studied in variety plants. In *Arabidopsis thaliana*, *AtNLP8* and *AtNLP9* are highly expressed in senescent leaves and seeds, but lower in other organs. In rice, *OsNLP1* and *OsNLP3* are preferentially expressed in the source organs (Schauser et al., 2005). Similarly, in this study, *CnD-NLP2* and *CnT-NLP2/5* showed similar expression patterns across tissues, with most other genes exhibiting higher expression in leaves than in roots and petioles. Specifically, *CnD-NLP3* and *CnT-NLP1* were highly expressed in young leaves, while *CnD-NLP1/4/5/6/7* and *CnT-NLP3/4/6* were highly expressed in mature leaves (**Figures 6a, b**), indicating that these genes may be involved in leaves growth and development. In addition, lower *NLP* expression in coconut roots compared to leaf tissues suggests a primary role in nitrate transport rather than absorption (Kumar et al., 2018). In addition, in the study of the *MeNLP* gene of tropical crop cassava, it was found that the number of members of the *MeNLP* gene family was similar to that of *CnNLP*, but *MeNLP* was mainly expressed in root, which may be caused by species differences (Lin et al., 2023).

In the NLP protein interaction network map of coconut, AtNLP7/8 interacted with the nitrate regulatory protein AtNRG2 and transcription factors AtHRS1 and AtLBD37 (**Figure 2d**). Our results indicated that CnD-NLP3/4/5 and CnT-NLP3/4/5, which are homologous to AtNLP7/8 in coconut, may also participate in nitrate response. In addition, we analyzed the promoters of the *NLP* gene family members in coconut and identified several abiotic stress responses *cis*-acting elements, suggesting that *NLP* gene may be regulated by these *cis*-acting elements in their promoter under abiotic stress conditions (**Supplementary Figure S3**). Studies have shown that *OsNLP1*, a key gene for nitrogen utilization in rice, is rapidly induced under nitrogen starvation (Alfatih et al., 2020). Moreover, previous research indicated that *Arabidopsis thaliana* lacking *AtNLP7* exhibited greater drought and salt tolerance than the wild type (Castaings et al., 2009; Le et al., 2022), suggesting that the expression of *NLP* genes affects drought and salt tolerance in plants. In this study, it was observed that under nitrogen deficiency, drought, and salt stress, the expression of *CnD-NLP5* gene in young leaves continuously increased under nitrogen deficiency stress, while the expression of other genes in different tissues initially increased and then decreased, peaking at a specific time points. Under nitrogen deficiency stress, *CnD-NLP5* expression in petioles and leaves changed significantly, indicating that its potential as a key gene in nitrogen deficiency response. The expression of *CnD-NLP4/5/6* gene in roots changed significantly under drought stress, suggesting that these may be key genes in the drought stress response. Similarly, *CnD-NLP1* expression in petioles and leaves changed significantly under salt stress, indicating that it may be the key gene in response to salt stress (**Figure 7a**). These results indicate that the *NLP* gene family plays a key role in tissue development and stress response, which lays a foundation for further study of the function of *NLPs* in coconut.

## 5 Conclusions

Genome-wide analysis identified 7 *NLP* members in *Aromatic Dwarf* and 6 *NLP* members in *Hainan Tall*. Bioinformatics analysis revealed the physicochemical properties, protein structure, protein interaction, conserved motifs, gene structure, and evolutionary relationship of these *NLP* members. Functional predictions indicated that these *NLP* genes are closely related to responses to abiotic stress. Tissue expression profile analysis showed that *CnD-NLPs* were mainly expressed in leaves. Stress response analysis showed that *NLP* transcription factors regulate responses to nitrogen deficiency, drought, and salt stress. Specifically, *CnD-NLP5* may be a key gene in response to nitrogen deficiency, *CnD-NLP4/5/6* may be a key gene in response to drought stress, and *CnD-NLP1* may be a key gene in response to salt stress. These findings lay a foundation for further study of the function of *NLP* gene family in coconut.

## Data Availability

The original contributions presented in the study are included in the article/**Supplementary Material**. Further inquiries can be directed to the corresponding authors.
