# Supplementary material for: Based on two coconut (Cocos nucifera L.) genome-wide investigation of NODULE-INCEPTION-like protein family: evolution and expression profiles during development and stress
Source: Front Plant Sci. 2025 Apr 16;16:1565559. doi: 10.3389/fpls.2025.1565559 (PMC12040979; doi:10.3389/fpls.2025.1565559)
Supplement: Supplementary file 1 [file DataSheet1.docx]

Supplementary Material

# Supplementary Figures


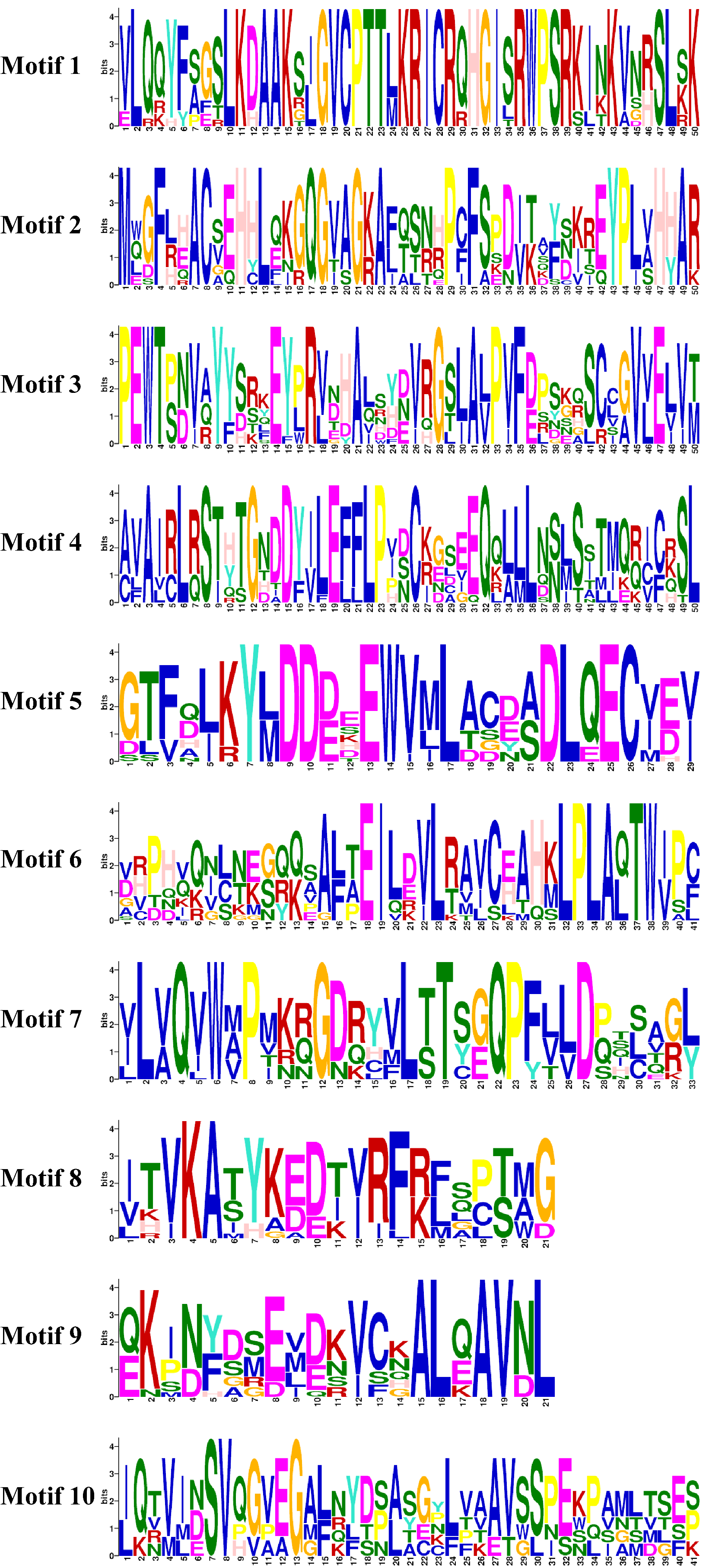


**Figure S1.** Sequence logos of the 10 conserved motifs.


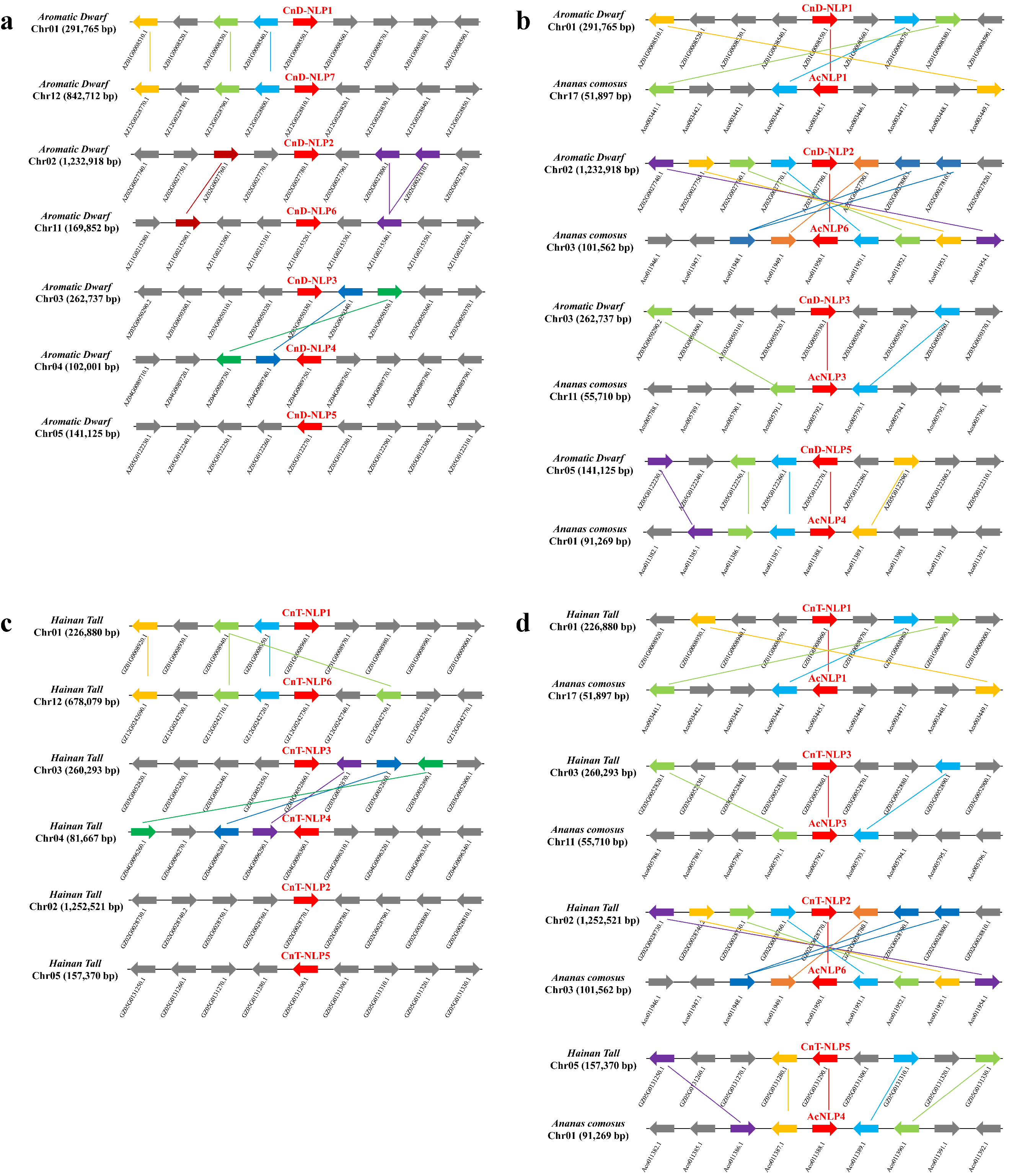


**Figure S2.** Syntenic analysis of *CnNLP* genes. The lines represent collinear genes, with red lines indicating the collinearity relationships specific to *NLP* genes. A left-facing arrow denotes the reverse orientation of the gene on the corresponding chromosome, while a right-facing arrow signifies the forward orientation. (a) Syntenic analysis of *CnD-NLP* genes. (b) Syntenic analysis of *NLP* genes in *Aromatic Dwarf* and *Ananas comosus*. (c) Syntenic analysis of *CnT-NLP* genes. (d) Syntenic analysis of *NLP* genes in *Hainan Tall* and *Ananas comosus*.


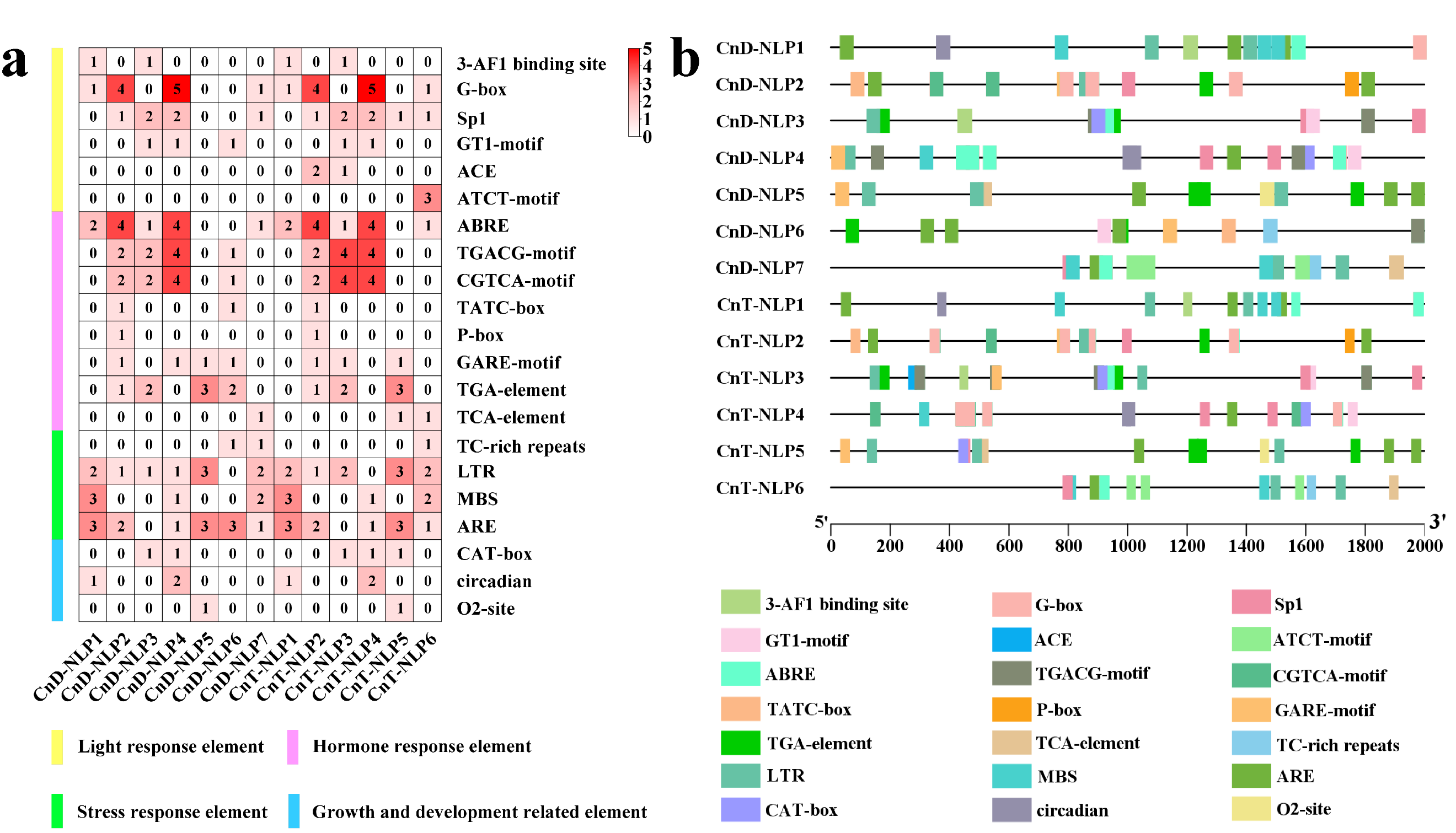


**Figure S3.** Prediction of *cis*-acting elements of *NLP* promoter in Coconut. (a) Number of cis-acting elements. (b) The position of the cis-acting elements.

# Supplementary Tables

**Table S1.** Primers for qRT-PCR.

| Gene name | Forward primer sequence (5′-3′) | Reverse primer sequence (5′-3′) |
| --- | --- | --- |
| *β-actin* | ATAAAGTATGGCTGATGCTGAGG | CAACAATGCTTGGGAACACA |
| *CnD/T-NLP1* | GGACCATTACAGCAGGCTAACATTG | TGACACTGAAGGCTCCGTAAACTC |
| *CnD/T-NLP2* | TTAGGTGTTGTGGAGGTGGTGATG | AGATTAACAGCCTGAAGAGCATTGC |
| *CnD/T-NLP3* | GTATCCTCGCCTTACCCATGCC | GCTCAACCACACCAACACAAGAC |
| *CnD/T-NLP4* | CCTCGCTGACTTGTCCACTTCC | AACTTTGGCACCCTCGCTCTG |
| *CnD/T-NLP5* | GTGGAAGATTGGAAGAAGAGGCTTG | CCGTGATGATGAAGTTGTGGTTGG |
| *CnD-NLP6* | CACCTATTGAGCGTTGTTCTTCTGG | GCAACCGAAGCCGAACTTTCTC |
| *CnD/T-NLP7/6* | GGAGTGGAGGGAGCATTAAGGTATG | AACATCAGGAGCAGAAGACACAGG |

**Table S2.** Secondary structure of NLP protein in coconut.

| Protein | Alpha helix (aa)  (Proportion (%)) | Extended strand (aa)  (Proportion (%)) | Beta turn (aa)  (Proportion (%)) | Random coil (aa)  (Proportion (%)) |
| --- | --- | --- | --- | --- |
| CnD-NLP1 | 203 (27.66) | 57 (7.77) | 13 (1.77) | 461 (62.81) |
| CnD-NLP2 | 262 (27.41) | 59 (6.17) | 8 (0.84) | 627 (65.59) |
| CnD-NLP3 | 253 (26.55) | 71 (7.45) | 14 (1.47) | 615 (64.53) |
| CnD-NLP4 | 273 (28.53) | 71 (7.42) | 15 (1.57) | 598 (62.49) |
| CnD-NLP5 | 230 (25.70) | 67 (7.49) | 12 (1.34) | 586 (65.47) |
| CnD-NLP6 | 247 (26.85) | 67 (7.28) | 14 (1.52) | 592 (64.35) |
| CnD-NLP7 | 235 (24.50) | 75 (7.82) | 10 (1.04) | 639 (66.63) |
| CnT-NLP1 | 203 (27.66) | 57 (7.77) | 13 (1.77) | 461 (62.81) |
| CnT-NLP2 | 254 (26.57) | 68 (7.11) | 10 (1.05) | 624 (65.27) |
| CnT-NLP3 | 253 (26.55) | 71 (7.45) | 14 (1.47) | 615 (64.53) |
| CnT-NLP4 | 273 (28.53) | 71 (7.42) | 15 (1.57) | 598 (62.49) |
| CnT-NLP5 | 230 (25.70) | 67 (7.49) | 12 (1.34) | 586 (65.47) |
| CnT-NLP6 | 235 (24.50) | 75 (7.82) | 10 (1.04) | 639 (66.63) |

**Table S3.** Details of the predicted protein tertiary structure.

| Gene Name | GMQE | Seq Identity | Oligo-State | Template | Templete Description |
| --- | --- | --- | --- | --- | --- |
| CnD-NLP1 | 0.62 | 78.04% | Monomer | A0A6I9RQB9.1.A | Protein NLP2 |
| CnD-NLP2 | 0.59 | 90.66% | Monomer | A0A6I9R0J5.1.A | Protein NLP1 isoform X1 |
| CnD-NLP3 | 0.59 | 92.11% | Monomer | A0A6I9QQG2.1.A | LOW QUALITY PROTEIN: protein NLP3 |
| CnD-NLP4 | 0.59 | 90.14% | Monomer | A0A6I9QVQ6.1.A | Protein NLP3 |
| CnD-NLP5 | 0.61 | 86.47% | Monomer | A0A6I9QG16.1.A | Protein NLP2 |
| CnD-NLP6 | 0.60 | 91.85% | Monomer | A0A6I9S130.1.A | Protein NLP1 |
| CnD-NLP7 | 0.60 | 92.08% | Monomer | A0A6I9RQB9.1.A | Protein NLP2 |
| CnT-NLP1 | 0.62 | 78.04% | Monomer | A0A6I9RQB9.1.A | Protein NLP2 |
| CnT-NLP2 | 0.59 | 90.98% | Monomer | A0A6I9R0J5.1.A | Protein NLP1 isoform X1 |
| CnT-NLP3 | 0.59 | 92.11% | Monomer | A0A6I9QQG2.1.A | LOW QUALITY PROTEIN: protein NLP3 |
| CnT-NLP4 | 0.59 | 90.14% | Monomer | A0A6I9QVQ6.1.A | Protein NLP3 |
| CnT-NLP5 | 0.61 | 86.47% | Monomer | A0A6I9QG16.1.A | Protein NLP2 |
| CnT-NLP6 | 0.60 | 92.08% | Monomer | A0A6I9RQB9.1.A | Protein NLP2 |

**Table S4.** String mapping.

| Query Index | Query Item | String Id | Identity | Bit score | E-value | Preferred Name | Annotation |
| --- | --- | --- | --- | --- | --- | --- | --- |
| **1** | **CnD-NLP1** | **3702.Q9M1B0** | **47.4** | **559.3** | **3.50e-159** | **AtNLP9** | **Protein AtNLP9; Probable transcription factor.** |
| 1 | CnD-NLP1 | 3702.O22864 | 45.8 | 546.2 | 3.10e-155 | AtNLP8 | Protein AtNLP8; Probable transcription factor. |
| 1 | CnD-NLP1 | 3702.Q84TH9 | 34.9 | 363.6 | 2.80e-100 | AtNLP7 | Protein AtNLP7; Transcription factor involved in regulation of nitrate assimilation and in transduction of the nitrate signal. |
| 1 | CnD-NLP1 | 3702.Q8RWY4 | 33.2 | 352.4 | 6.50e-97 | AtNLP6 | Protein AtNLP6; Probable transcription factor. |
| 1 | CnD-NLP1 | 3702.Q9SFW8 | 32.2 | 311.2 | 1.70e-84 | AtNLP5 | Protein AtNLP5; Probable transcription factor. |
| **2** | **CnD-NLP2** | **3702.Q9LE38** | **43.9** | **617.5** | **1.40e-176** | **AtNLP4** | **Protein AtNLP4; Probable transcription factor.** |
| 2 | CnD-NLP2 | 3702.Q9SFW8 | 40.6 | 611.3 | 1.00e-174 | AtNLP5 | Protein AtNLP5; Probable transcription factor. |
| 2 | CnD-NLP2 | 3702.Q7X9B9 | 44.2 | 604 | 1.60e-172 | AtNLP2 | Protein AtNLP2; Probable transcription factor. |
| 2 | CnD-NLP2 | 3702.Q8H111 | 41 | 584.7 | 1.00e-166 | AtNLP1 | Protein AtNLP1; Probable transcription factor. |
| 2 | CnD-NLP2 | 3702.Q84TH9 | 34.7 | 452.6 | 6.10e-127 | AtNLP7 | Protein AtNLP7; Transcription factor involved in regulation of nitrate assimilation and in transduction of the nitrate signal. |
| **3** | **CnD-NLP3** | **3702.Q84TH9** | **48.9** | **816.2** | **2.10e-236** | **AtNLP7** | **Protein AtNLP7; Transcription factor involved in regulation of nitrate assimilation and in transduction of the nitrate signal.** |
| 3 | CnD-NLP3 | 3702.Q8RWY4 | 46.7 | 748.8 | 4.10e-216 | AtNLP6 | Protein AtNLP6; Probable transcription factor. |
| 3 | CnD-NLP3 | 3702.O22864 | 35.5 | 463.4 | 3.40e-130 | AtNLP8 | Protein AtNLP8; Probable transcription factor. |
| 3 | CnD-NLP3 | 3702.Q9M1B0 | 36.9 | 457.6 | 1.90e-128 | AtNLP9 | Protein AtNLP9; Probable transcription factor. |
| 3 | CnD-NLP3 | 3702.Q9SFW8 | 35.7 | 452.2 | 7.90e-127 | AtNLP5 | Protein AtNLP5; Probable transcription factor. |
| **4** | **CnD-NLP4** | **3702.Q84TH9** | **48.3** | **809.7** | **2.00e-234** | **AtNLP7** | **Protein AtNLP7; Transcription factor involved in regulation of nitrate assimilation and in transduction of the nitrate signal.** |
| 4 | CnD-NLP4 | 3702.Q8RWY4 | 49.1 | 772.7 | 2.60e-223 | AtNLP6 | Protein AtNLP6; Probable transcription factor. |
| 4 | CnD-NLP4 | 3702.Q9SFW8 | 37.2 | 458.4 | 1.10e-128 | AtNLP5 | Protein AtNLP5; Probable transcription factor. |
| 4 | CnD-NLP4 | 3702.Q9LE38 | 35.9 | 446.4 | 4.30e-125 | AtNLP4 | Protein AtNLP4; Probable transcription factor. |
| 4 | CnD-NLP4 | 3702.Q9M1B0 | 35.3 | 435.6 | 7.70e-122 | AtNLP9 | Protein AtNLP9; Probable transcription factor. |
| **5** | **CnD-NLP5** | **3702.O22864** | **40.4** | **584.7** | **9.60e-167** | **AtNLP8** | **Protein AtNLP8; Probable transcription factor.** |
| 5 | CnD-NLP5 | 3702.Q9M1B0 | 44.2 | 583.6 | 2.10e-166 | AtNLP9 | Protein AtNLP9; Probable transcription factor. |
| 5 | CnD-NLP5 | 3702.Q9SFW8 | 35.3 | 400.6 | 2.60e-111 | AtNLP5 | Protein AtNLP5; Probable transcription factor. |
| 5 | CnD-NLP5 | 3702.Q9LE38 | 34.4 | 384.4 | 1.90e-106 | AtNLP4 | Protein AtNLP4; Probable transcription factor. |
| 5 | CnD-NLP5 | 3702.Q84TH9 | 33.1 | 380.9 | 2.10e-105 | AtNLP7 | Protein AtNLP7; Transcription factor involved in regulation of nitrate assimilation and in transduction of the nitrate signal. |
| **6** | **CnD-NLP6** | **3702.Q9SFW8** | **41.8** | **617.5** | **1.40e-176** | **AtNLP5** | **Protein AtNLP5; Probable transcription factor.** |
| 6 | CnD-NLP6 | 3702.Q9LE38 | 45.8 | 616.3 | 3.00e-176 | AtNLP4 | Protein AtNLP4; Probable transcription factor. |
| 6 | CnD-NLP6 | 3702.Q7X9B9 | 42.1 | 598.6 | 6.60e-171 | AtNLP2 | Protein AtNLP2; Probable transcription factor. |
| 6 | CnD-NLP6 | 3702.Q8H111 | 40.7 | 582.4 | 4.90e-166 | AtNLP1 | Protein AtNLP1; Probable transcription factor. |
| 6 | CnD-NLP6 | 3702.Q84TH9 | 35.3 | 465.3 | 8.70e-131 | AtNLP7 | Protein AtNLP7; Transcription factor involved in regulation of nitrate assimilation and in transduction of the nitrate signal. |
| **7** | **CnD-NLP7** | **3702.Q9M1B0** | **49.7** | **693.3** | **2.00e-199** | **AtNLP9** | **Protein AtNLP9; Probable transcription factor.** |
| 7 | CnD-NLP7 | 3702.O22864 | 47.1 | 676 | 3.40e-194 | AtNLP8 | Protein AtNLP8; Probable transcription factor. |
| 7 | CnD-NLP7 | 3702.Q84TH9 | 35.6 | 420.6 | 2.60e-117 | AtNLP7 | Protein AtNLP7; Transcription factor involved in regulation of nitrate assimilation and in transduction of the nitrate signal. |
| 7 | CnD-NLP7 | 3702.Q8RWY4 | 33.7 | 398.3 | 1.40e-110 | AtNLP6 | Protein AtNLP6; Probable transcription factor. |
| 7 | CnD-NLP7 | 3702.Q9SFW8 | 34.1 | 392.9 | 5.70e-109 | AtNLP5 | Protein AtNLP5; Probable transcription factor. |
| **8** | **CnT-NLP1** | **3702.Q9M1B0** | **47.4** | **559.3** | **3.50e-159** | **AtNLP9** | **Protein AtNLP9; Probable transcription factor.** |
| 8 | CnT-NLP1 | 3702.O22864 | 45.8 | 546.2 | 3.10e-155 | AtNLP8 | Protein AtNLP8; Probable transcription factor. |
| 8 | CnT-NLP1 | 3702.Q84TH9 | 34.9 | 363.6 | 2.80e-100 | AtNLP7 | Protein AtNLP7; Transcription factor involved in regulation of nitrate assimilation and in transduction of the nitrate signal. |
| 8 | CnT-NLP1 | 3702.Q8RWY4 | 33.2 | 352.4 | 6.50e-97 | AtNLP6 | Protein AtNLP6; Probable transcription factor. |
| 8 | CnT-NLP1 | 3702.Q9SFW8 | 32.2 | 311.2 | 1.70e-84 | AtNLP5 | Protein AtNLP5; Probable transcription factor. |
| **9** | **CnT-NLP2** | **3702.Q9LE38** | **44.0** | **618.2** | **8.30e-177** | **AtNLP4** | **Protein AtNLP4; Probable transcription factor.** |
| 9 | CnT-NLP2 | 3702.Q9SFW8 | 40.9 | 618.2 | 8.30e-177 | AtNLP5 | Protein AtNLP5; Probable transcription factor. |
| 9 | CnT-NLP2 | 3702.Q7X9B9 | 44.4 | 608.6 | 6.60e-174 | AtNLP2 | Protein AtNLP2; Probable transcription factor. |
| 9 | CnT-NLP2 | 3702.Q8H111 | 40.9 | 587 | 2.10e-167 | AtNLP1 | Protein AtNLP1; Probable transcription factor. |
| 9 | CnT-NLP2 | 3702.Q84TH9 | 34.7 | 451.1 | 1.80e-126 | AtNLP7 | Protein AtNLP7; Transcription factor involved in regulation of nitrate assimilation and in transduction of the nitrate signal. |
| **10** | **CnT-NLP3** | **3702.Q84TH9** | **48.9** | **816.2** | **2.10e-236** | **AtNLP7** | **Protein AtNLP7; Transcription factor involved in regulation of nitrate assimilation and in transduction of the nitrate signal.** |
| 10 | CnT-NLP3 | 3702.Q8RWY4 | 46.7 | 748.8 | 4.10e-216 | AtNLP6 | Protein AtNLP6; Probable transcription factor. |
| 10 | CnT-NLP3 | 3702.O22864 | 35.5 | 463.4 | 3.40e-130 | AtNLP8 | Protein AtNLP8; Probable transcription factor. |
| 10 | CnT-NLP3 | 3702.Q9M1B0 | 36.9 | 457.6 | 1.90e-128 | AtNLP9 | Protein AtNLP9; Probable transcription factor. |
| 10 | CnT-NLP3 | 3702.Q9SFW8 | 35.7 | 452.2 | 7.90e-127 | AtNLP5 | Protein AtNLP5; Probable transcription factor. |
| **11** | **CnT-NLP4** | **3702.Q84TH9** | **48.3** | **809.7** | **2.00e-234** | **AtNLP7** | **Protein AtNLP7; Transcription factor involved in regulation of nitrate assimilation and in transduction of the nitrate signal.** |
| 11 | CnT-NLP4 | 3702.Q8RWY4 | 49.1 | 772.7 | 2.60e-223 | AtNLP6 | Protein AtNLP6; Probable transcription factor. |
| 11 | CnT-NLP4 | 3702.Q9SFW8 | 37.2 | 458.4 | 1.10e-128 | AtNLP5 | Protein AtNLP5; Probable transcription factor. |
| 11 | CnT-NLP4 | 3702.Q9LE38 | 35.9 | 446.4 | 4.30e-125 | AtNLP4 | Protein AtNLP4; Probable transcription factor. |
| 11 | CnT-NLP4 | 3702.Q9M1B0 | 35.3 | 435.6 | 7.70e-122 | AtNLP9 | Protein AtNLP9; Probable transcription factor. |
| **12** | **CnT-NLP5** | **3702.O22864** | **40.4** | **584.7** | **9.60e-167** | **AtNLP8** | **Protein AtNLP8; Probable transcription factor.** |
| 12 | CnT-NLP5 | 3702.Q9M1B0 | 44.2 | 583.6 | 2.10e-166 | AtNLP9 | Protein AtNLP9; Probable transcription factor. |
| 12 | CnT-NLP5 | 3702.Q9SFW8 | 35.3 | 400.6 | 2.60e-111 | AtNLP5 | Protein AtNLP5; Probable transcription factor. |
| 12 | CnT-NLP5 | 3702.Q9LE38 | 34.4 | 384.4 | 1.90e-106 | NAtLP4 | Protein AtNLP4; Probable transcription factor. |
| 12 | CnT-NLP5 | 3702.Q84TH9 | 33.1 | 380.9 | 2.10e-105 | AtNLP7 | Protein AtNLP7; Transcription factor involved in regulation of nitrate assimilation and in transduction of the nitrate signal. |
| **13** | **CnT-NLP6** | **3702.Q9M1B0** | **49.7** | **693.3** | **2.00e-199** | **AtNLP9** | **Protein AtNLP9; Probable transcription factor.** |
| 13 | CnT-NLP6 | 3702.O22864 | 47.1 | 676 | 3.40e-194 | AtNLP8 | Protein AtNLP8; Probable transcription factor. |
| 13 | CnT-NLP6 | 3702.Q84TH9 | 35.6 | 420.6 | 2.60e-117 | AtNLP7 | Protein AtNLP7; Transcription factor involved in regulation of nitrate assimilation and in transduction of the nitrate signal. |
| 13 | CnT-NLP6 | 3702.Q8RWY4 | 33.7 | 398.3 | 1.40e-110 | AtNLP6 | Protein AtNLP6; Probable transcription factor. |
| 13 | CnT-NLP6 | 3702.Q9SFW8 | 34.1 | 392.9 | 5.70e-109 | AtNLP5 | Protein AtNLP5; Probable transcription factor. |

Note: The bold parts in the table represents the *NLP* genes corresponding to coconut and *Arabidopsis* that were selected through a comprehensive analysis of identity, bit score, and e-value.

**Table S5.** Accession numbers of gene.

| Species | Name | Accession numbers |
| --- | --- | --- |
| *Elaeis guineensis* | *EgNLP1* | XP_010908915.1 |
| *Elaeis guineensis* | *EgNLP2* | XP_010912370.1 |
| *Elaeis guineensis* | *EgNLP3* | XP_010912769.1 |
| *Elaeis guineensis* | *EgNLP4* | XP_010915986.1 |
| *Elaeis guineensis* | *EgNLP5* | XP_010918248.1 |
| *Elaeis guineensis* | *EgNLP6* | XP_010918249.1 |
| *Elaeis guineensis* | *EgNLP7* | XP_010918250.1 |
| *Elaeis guineensis* | *EgNLP8* | XP_010918251.1 |
| *Elaeis guineensis* | *EgNLP9* | XP_010930267.1 |
| *Elaeis guineensis* | *EgNLP10* | XP_010935286.1 |
| *Elaeis guineensis* | *EgNLP11* | XP_010935287.1 |
| *Phoenix dactylifera* | *PdNLP1* | PDK_30s1064101g001 |
| *Phoenix dactylifera* | *PdNLP2* | PDK_30s1129411g009 |
| *Phoenix dactylifera* | *PdNLP3* | PDK_30s710451g012 |
| *Phoenix dactylifera* | *PdNLP4* | PDK_30s735141g001 |
| *Phoenix dactylifera* | *PdNLP5* | PDK_30s741891g011 |
| *Phoenix dactylifera* | *PdNLP6* | PDK_30s743291g002 |
| *Phoenix dactylifera* | *PdNLP7* | PDK_30s883821g007 |
| *Phoenix dactylifera* | *PdNLP8* | PDK_30s966651g002 |
| *Spirodela polyrhiza* | *SpNLP1* | Spipo11G0046200 |
| *Spirodela polyrhiza* | *SpNLP2* | Spipo13G0014600 |
| *Spirodela polyrhiza* | *SpNLP3* | Spipo14G0026000 |
| *Spirodela polyrhiza* | *SpNLP4* | Spipo17G0032300 |
| *Ananas comosus* | *AcNLP1* | Aco003445.1 |
| *Ananas comosus* | *AcNLP2* | Aco004459.1 |
| *Ananas comosus* | *AcNLP3* | Aco005792.1 |
| *Ananas comosus* | *AcNLP4* | Aco011388.1 |
| *Ananas comosus* | *AcNLP5* | Aco011836.1 |
| *Ananas comosus* | *AcNLP6* | Aco011950.1 |
| *Ananas comosus* | *AcNLP7* | Aco018366.1 |
| *Ananas comosus* | *AcNLP8* | Aco028022.1 |
| *Musa acuminate* | *MaNLP1* | GSMUA_Achr1P18080_001 |
| *Musa acuminate* | *MaNLP2* | GSMUA_Achr2P05790_001 |
| *Musa acuminate* | *MaNLP3* | GSMUA_Achr3P03250_001 |
| *Musa acuminate* | *MaNLP4* | GSMUA_Achr3P14760_001 |
| *Musa acuminate* | *MaNLP5* | GSMUA_Achr3P25240_001 |
| *Musa acuminate* | *MaNLP6* | GSMUA_Achr5P19140_001 |
| *Musa acuminate* | *MaNLP7* | GSMUA_Achr6P28700_001 |
| *Musa acuminate* | *MaNLP8* | GSMUA_Achr8P04280_001 |
| *Musa acuminate* | *MaNLP9* | GSMUA_Achr8P28910_001 |
| *Musa acuminate* | *MaNLP10* | GSMUA_Achr11P22400_001 |
| *Musa acuminate* | *MaNLP11* | GSMUA_AchrUn_randomP00660_001 |
| *Vitis vinifera* | *VvNLP1* | GSVIVT01013370001 |
| *Vitis vinifera* | *VvNLP2* | GSVIVT01019600001 |
| *Vitis vinifera* | *VvNLP3* | GSVIVT01023886001 |
| *Vitis vinifera* | *VvNLP4* | GSVIVT01038090001 |
| *Pyunus persica* | *PpNLP1* | Prupe.1G200100.1.p |
| *Pyunus persica* | *PpNLP2* | Prupe.5G011200.1.p |
| *Pyunus persica* | *PpNLP3* | Prupe.5G125000.1.p |
| *Pyunus persica* | *PpNLP4* | Prupe.8G178400.1.p |
| *Theobroma cacao* | *TcNLP1* | Thecc1EG006453t1 |
| *Theobroma cacao* | *TcNLP2* | Thecc1EG016510t1 |
| *Theobroma cacao* | *TcNLP3* | Thecc1EG019949t1 |
| *Theobroma cacao* | *TcNLP4* | Thecc1EG034391t1 |
| *Oryza sativa* | *OsNLP1* | LOC_Os03g03900.1 |
| *Oryza sativa* | *OsNLP2* | LOC_Os04g41850.1 |
| *Oryza sativa* | *OsNLP3* | LOC_Os01g13540.1 |
| *Oryza sativa* | *OsNLP4* | LOC_Os09g37710.2 |
| *Oryza sativa* | *OsNLP5* | LOC_Os11g16290.1 |
| *Oryza sativa* | *OsNLP6* | LOC_Os02g04340.1 |
| *Arabidopsis thaliana* | *AtNLP1* | AT2G17150.1 |
| *Arabidopsis thaliana* | *AtNLP2* | AT4G35270.4 |
| *Arabidopsis thaliana* | *AtNLP3* | AT4G38340.1 |
| *Arabidopsis thaliana* | *AtNLP4* | AT1G20640.2 |
| *Arabidopsis thaliana* | *AtNLP5* | AT1G76350.1 |
| *Arabidopsis thaliana* | *AtNLP6* | AT1G64530.1 |
| *Arabidopsis thaliana* | *AtNLP7* | AT4G24020.1 |
| *Arabidopsis thaliana* | *AtNLP8* | AT2G43500.5 |
| *Arabidopsis thaliana* | *AtNLP9* | AT3G59580.2 |
| *Sorghum bicolor* | *SbNLP1* | Sobic.001G517800.1.p |
| *Sorghum bicolor* | *SbNLP2* | Sobic.002G287400.1.p |
| *Sorghum bicolor* | *SbNLP3* | Sobic.003G003600.1.p |
| *Sorghum bicolor* | *SbNLP4* | Sobic.004G034100.1.p |
| *Sorghum bicolor* | *SbNLP5* | Sobic.006G133900.1.p |
| *Glycine max* | *GmNLP1* | Glyma.04G017400.1.p |
| *Glycine max* | *GmNLP2* | Glyma.06G017800.1.p |
| *Glycine max* | *GmNLP3* | Glyma.09G137000.1.p |
| *Glycine max* | *GmNLP4* | Glyma.10G234100.1.p |
| *Glycine max* | *GmNLP5* | Glyma.11G125500.1.p |
| *Glycine max* | *GmNLP6* | Glyma.12G050100.1.p |
| *Glycine max* | *GmNLP7* | Glyma.13G346300.1.p |
| *Glycine max* | *GmNLP8* | Glyma.15G027900.1.p |
| *Glycine max* | *GmNLP9* | Glyma.16G182400.1.p |
| *Glycine max* | *GmNLP10* | Glyma.20G160200.1.p |
| *Populus trichocarpa* | *PtNLP1* | Potri.001G087900.1 |
| *Populus trichocarpa* | *PtNLP2* | Potri.001G293300.1 |
| *Populus trichocarpa* | *PtNLP3* | Potri.002G009700.1 |
| *Populus trichocarpa* | *PtNLP4* | Potri.003G143000.1 |
| *Populus trichocarpa* | *PtNLP5* | Potri.004G205500.1 |
| *Populus trichocarpa* | *PtNLP6* | Potri.005G251700.1 |
| *Populus trichocarpa* | *PtNLP7* | Potri.007G133400.1 |
| *Populus trichocarpa* | *PtNLP8* | Potri.009G087500.1 |
| *Populus trichocarpa* | *PtNLP9* | Potri.009G166400.1 |
| *Populus trichocarpa* | *PtNLP10* | Potri.009G166800.1 |
| *Populus trichocarpa* | *PtNLP11* | Potri.009G166900.1 |
| *Populus trichocarpa* | *PtNLP12* | Potri.016G042900.1 |
| *Populus trichocarpa* | *PtNLP13* | Potri.017G018500.1 |
| *Populus trichocarpa* | *PtNLP14* | Potri.T078200.1 |
| *Cocos nucifera L. cv. Aromatic Dwarf* | *CnD-NLP1* | AZ01G0008550.1 |
| *Cocos nucifera L. cv. Aromatic Dwarf* | *CnD-NLP2* | AZ02G0027780.1 |
| *Cocos nucifera L. cv. Aromatic Dwarf* | *CnD-NLP3* | AZ03G0050330.1 |
| *Cocos nucifera L. cv. Aromatic Dwarf* | *CnD-NLP4* | AZ04G0089750.1 |
| *Cocos nucifera L. cv. Aromatic Dwarf* | *CnD-NLP5* | AZ05G0122270.1 |
| *Cocos nucifera L. cv. Aromatic Dwarf* | *CnD-NLP6* | AZ11G0215320.1 |
| *Cocos nucifera L. cv. Aromatic Dwarf* | *CnD-NLP7* | AZ12G0228810.1 |
| *Cocos nucifera L. cv. Hainan Tall* | *CnT-NLP1* | GZ01G0008960.1 |
| *Cocos nucifera L. cv. Hainan Tall* | *CnT-NLP2* | GZ02G0028770.1 |
| *Cocos nucifera L. cv. Hainan Tall* | *CnT-NLP3* | GZ03G0052860.1 |
| *Cocos nucifera L. cv. Hainan Tall* | *CnT-NLP4* | GZ04G0096300.1 |
| *Cocos nucifera L. cv. Hainan Tall* | *CnT-NLP5* | GZ05G0131290.1 |
| *Cocos nucifera L. cv. Hainan Tall* | *CnT-NLP6* | GZ12G0242730.1 |

**Table S6.** Types of *NLP* gene *Ka/Ks* ratio in Coconut.

| Gene name | Gene name | *Ka* | *Ks* | *Ka/Ks* | Selection type |
| --- | --- | --- | --- | --- | --- |
| *CnD-NLP1* | *CnD-NLP5* | 0.342 | 1.207 | 0.284 | Purifying |
| *CnD-NLP1* | *CnD-NLP7* | 0.116 | 0.282 | 0.411 | Purifying |
| *CnD-NLP2* | *CnD-NLP6* | 0.280 | 0.953 | 0.293 | Purifying |
| *CnD-NLP5* | *CnD-NLP7* | 0.329 | 1.272 | 0.259 | Purifying |
| *CnT-NLP1* | *CnT-NLP5* | 0.342 | 1.218 | 0.281 | Purifying |
| *CnT-NLP1* | *CnT-NLP6* | 0.116 | 0.282 | 0.411 | Purifying |
| *CnT-NLP3* | *CnT-NLP4* | 0.241 | 0.885 | 0.273 | Purifying |
| *CnT-NLP5* | *CnT-NLP6* | 0.329 | 1.281 | 0.257 | Purifying |
